# Impact of Protein Corona Formation on the Thermoresponsive Behavior of Acrylamide-Based Nanogels

**DOI:** 10.1021/acs.biomac.3c01405

**Published:** 2024-01-19

**Authors:** Federico Traldi, Marina Resmini

**Affiliations:** Department of Chemistry, SPCS, Queen Mary University of London, London E1 4NS, U.K.

## Abstract

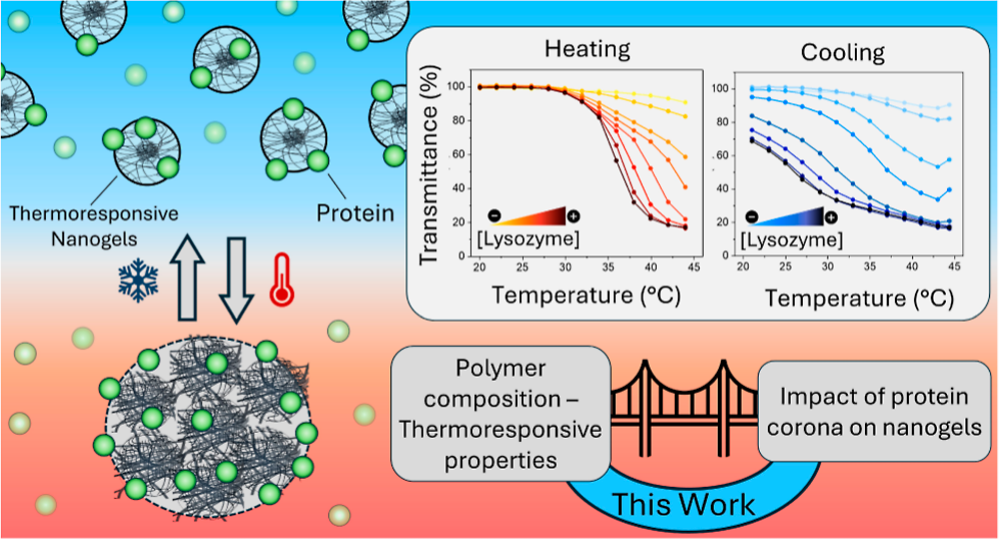

The ability to fine-tune the volume phase transition temperature (VPTT) of thermoresponsive nanoparticles is essential to their successful application in drug delivery. The rational design of these materials is limited by our understanding of the impact that nanoparticle–protein interactions have on their thermoresponsive behavior. In this work, we demonstrate how the formation of protein corona impacts the transition temperature values of acrylamide-based nanogels and their reversibility characteristics, in the presence of lysozyme, given its relevance for the ocular and intranasal administration route. Nanogels were synthesized with *N*-isopropylacrylamide or *N*-*n*-propylacrylamide as backbone monomers, methylenebis(acrylamide) (2.5–20 molar %) as a cross-linker, and functionalized with negatively charged monomers 2-acrylamido-2-methylpropanesulfonic acid, *N*-acryloyl-l-proline, or acrylic acid; characterization showed comparable particle diameter (*c.a*.10 nm), but formulation-dependent thermoresponsive properties, in the range 28–54 °C. Lysozyme was shown to form a complex with the negatively charged nanogels, lowering their VPTT values; the hydrophilic nature of the charged comonomer controlled the drop in VPTT upon complex formation, while matrix rigidity only had a small, yet significant effect. The cross-linker content was found to play a major role in determining the reversibility of the temperature-dependent transition of the complexes, with only 20 molar % cross-linked-nanogels displaying a fully reversible transition. These results demonstrate the importance of evaluating protein corona formation in the development of drug delivery systems based on thermoresponsive nanoparticles.

## Introduction

Nanomaterials are widely studied for their potential applications in medicine, in particular as drug delivery systems, designed to improve bioavailability,^1^ targeting,^2^ and to limit side effects.^3^ Covalently cross-linked nanogels are an interesting material, combining properties of hydrogels with nanoparticles, and offering advantages such as very small particle size, swelling behavior and high colloidal stability,^4^ high drug loading capacity,^5^ and easy-to-tailor formulation, which allows us to introduce stimuli-responsive properties such as temperature,^6^ pH,^7^ redox potential,^8^ ionic strength,^9^ and others.^10^

Thermoresponsive nanogels are attractive due to the physiological relevance of temperature for drug delivery; these materials undergo conformational changes in response to temperature variations, which can be used, for instance, to release cargos in carcinogenic or inflamed tissues, in applications like cancer treatment,^11^ neurological disorders,^12^ and gene therapy.^13^ Both local and systemic administration routes have been explored, with ocular and intranasal showing promise.^14−17^ In addition, the incorporation of charged comonomers in the formulation allows fine-tuning of the transition temperature to suit specific applications, yielding nanogels with enhanced drug encapsulation capacity,^18^ easier permeation through biological barriers,^19,20^ and dual thermo-pH-responsive properties;^21,22^ these advantages make charged nanogels particularly appealing materials for applications in drug delivery.

Studies have demonstrated that nanoparticles, when in contact with biological media, can interact with proteins, via hydrophobic and electrostatic interactions,^23^ resulting in complex formation (protein corona) and alterations of their physicochemical properties. The protein corona has been shown to change the biological behavior of nanomaterials in terms of toxicity,^24,25^ bioavailability,^26,27^ and targeting,^28,29^ thus requiring careful evaluation when designing drug delivery systems.

When developing thermoresponsive nanogels that are covalently cross-linked, the volume phase transition temperature (VPTT) has been shown to drive protein corona formation, leading to larger particle size^30^ and aggregation,^31^ driven by the increased hydrophobicity of the polymers above their VPTT. Given the importance that the fine-tuning of VPTT has in the development of thermoresponsive nanoparticles, the influence of salts,^32^ solvents,^33^ and surfactants,^34^ on the transition temperature has been investigated. However, the effect of protein corona formation on the VPTT value has not been reported to the best of our knowledge, together with the impact of surface functional groups and matrix rigidity. This lack of knowledge limits the rational design of drug delivery systems with fine-tuned thermoresponsive properties in complex media, especially when targeting ocular, intranasal, and oral administration routes, where nanoparticle–protein interactions occur in the protein-rich mucus.

We previously reported the development of thermoresponsive nanogels as drug delivery vectors,^35^ demonstrating the role of chemical composition and synthetic methodologies on their thermoresponsive behavior, both in buffer solutions^9^ and at the air/water interface.^36,37^ Additionally, our studies on the environmental exposure of nanogels to zebrafish larvae showed the high toxicity of positively charged formulations of *N*-isopropylacrylamide (NIPAM)-based nanogels,^38^ which prompted us to focus on negatively charged nanogels for this study.

Here, we report the impact of protein–nanogel interactions on the thermoresponsive properties of a range of negatively charged nanogels, using NIPAM or *N*-*n*-propylacrylamide (NPAM) as backbone monomers and methylenebis(acrylamide) (MBA) as a cross-linker (2.5 and 20 molar %). Three different comonomers, *N*-acryloyl-l-proline (ProAM), acrylic acid (AA), and 2-acrylamido-2-methyl-1-propanesulfonic (AMPS) acid were included in the formulations to introduce different negative surface charges. The protein corona formation was evaluated using lysozyme (Lyso) as model protein, which presents an overall positive^39^ charge at physiological pH, and is highly concentrated in biological fluids such as saliva,^40^ tears,^41^ and airways mucus,^42^ making this study fundamental for the application of nanogels as oral,^43^ ocular,^14,15^ or intranasal^16,17^ drug delivery systems. Bovine serum albumin (BSA) was used as comparison, given its overall negative charge,^44^ allowing us to evaluate the potential effect of electrostatic interactions. The impact of nanogel–protein interactions on the thermoresponsive behavior of the nanogels was characterized using UV–vis spectroscopy, dynamic light scattering (DLS), and circular dichroism (CD), which also allowed us to study the reversibility of the thermoresponsive transition.

## Experimental Section

### Materials

All chemicals were used as received, unless otherwise stated. *N*,*N*′-methylene-bis(acrylamide) (MBA, 99%), AA stabilized with hydroquinone monomethyl ether (AA, >99%), 1,2,4,5-tetramethylbenzene (98%), *n*-propylamine (98%), and l-proline reagent plus (≥99%) were purchased from Sigma-Aldrich (Gillingham, UK). AMPS (98%) and acryloyl chloride (96% with 400 ppm of phenothiazine stabilizer) were purchased from Alfa Aesar (Heysham, UK). Anhydrous magnesium sulfate (MgSO_4_), KOH, and HCl (37%) were purchased from Fisher Scientific (UK). NIPAM was purchased from Sigma-Aldrich and used after recrystallization from *n*-hexane. 2,2′-Azobis(isobutyronitrile) (AIBN, 98%) was purchased from Sigma-Aldrich and used after recrystallization from methanol. NPAM^21^ and *N*-acryloyl-l-proline (ProAM)^21^ were synthesized according to methodologies reported in a previous work. Dry dimethyl sulfoxide (DMSO) was purchased from Goss Scientific (Crewe, UK), while deuterated DMSO (DMSO-*d*_6_) employed for NMR conversion studies was obtained from Cambridge Isotope Laboratories. Acetone was obtained from Fisher, while methanol and *n*-hexane were received from Honeywell. Chloroform and toluene were purchased from VWR. Cellulose dialysis membrane (molecular-weight cutoff: 3500 Da, width: 34 mm, and diameter: 22 mm) was purchased from Medicell International Ltd. (London, UK). BSA (code A7030, ≥98% and lysozyme from chicken egg white (Lyso, code L6876, ≥90%) were obtained from Sigma-Aldrich (Gillingham, UK). Polytetrafluoroethylene (PTFE) syringe filters with a pore size of 0.2 μm and poly(ether sulfone) (PES) syringe filters with pore sizes of 0.2 and 0.45 μm were obtained from Fisher Scientific (Loughborough, UK).

### Synthesis of Nanogels

Nanogels were synthesized via high dilution radical polymerization (HDRP) following our previously reported procedure.^45^ For a typical preparation consisting of 80 molar % NPAM and 20 molar % MBA (where “molar %” refers to the % of moles of a specific monomer of the total moles of monomers in the feed solution), NPAM (167.46 mg, 1.48 mmol) and MBA (57.04, 0.37 mmol) were dissolved in 10 mL of anhydrous DMSO. This yields a total monomer concentration (*C*_M_) of 2% (*w*/*w*) according to eq 1
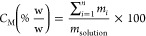
1where *m* is the mass and *n* is the total number of monomers, comonomers, and cross-linker (XL) (initiator excluded). The denominator refers to the total mass of the solution, including solvent, monomers, XL, and initiator. The initiator, AIBN (3.65 mg, 0.022 mmol), was added to the monomer solution in concentrations of 1 mol % of total moles of double bonds present in the mixture according to eq 2
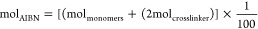
2The solution of monomers and the initiator was then transferred in a Wheaton bottle, and the vessel was sealed and purged with N_2_ for 15 min before being heated at 70 °C for 24 h. After 24 h, the reaction was quenched by letting the Wheaton bottle cool to room temperature and opening the sealed to let air in the vessel. The resulting nanogel clear colloidal solution in DMSO was purified via dialysis (MWCO 3500 Da, diameter 22 mm) against deionized water for 3 days changing water thrice a day. The purified nanogel solution in water was then freeze-dried (LTE Scientific Lyotrap), yielding a soft white powder that was stored at room temperature. Chemical yields were determined by weighing the dry polymer and subtracting the amount of the polymerization mixture withdrawn during the NMR conversion study (see Section 2.3).

### Quantification of Monomer Conversions by ^1^H NMR Spectroscopy

Monomer conversion studies were carried out by ^1^H NMR spectroscopy on each nanogel formulation. To do this, 250 μL of the polymerization mixture was drawn with a microsyringe immediately after complete dissolution of the monomers, XL, and initiator in DMSO (time zero, *t*_0h_) and after the reaction was quenched (time 24 h, *t*_24h_). These two aliquots were separately mixed with 250 μL of 1,2,4,5-tetramethylbenzene stock solution (8 mg/mL) in DMSO-*d*_6_ as the internal standard and transferred into an NMR tube. ^1^H NMR spectra were recorded in the solvent suppression mode at 298 K using a Bruker HD400, or Bruker AVIII400 spectrometer (400 MHz). The spectra were processed with Mestrenova software (version 6.0.2–5475). The acquired ^1^H NMR spectra were phased, baseline corrected, and integrated identically. The monomer conversion of each monomer was obtained by comparing the signals at *t*_0h_ and *t*_24h_ monomer peaks at 5.55 ppm (NIPAM), 5.57 ppm (NPAM), 5.63 ppm (MBA), 5.68 ppm (ProAM), 5.86 ppm (AA), and 5.51 ppm (AMPS) against the intensities of peaks of the internal standard at 6.88 ppm (1,2,4,5-tetramethylbenzene). Total monomer conversion was obtained in the same way by comparing the sums of all monomer signals at *t*_0h_ and *t*_24h_.

### Dynamic Light Scattering

*D*_h_ measurements were obtained by DLS using a Zetasizer Nano Ultra instrument operated with software ZS Xplorer (version 1.5.0.163) (Malvern Instruments Ltd., Malvern, UK). Nanogel stock colloidal solutions (1 mg/mL) were obtained by dissolving the dry nanogel powder in phosphate-buffered saline (PBS) (10 mM, pH 7.4), followed by sonication for 10 min and filtration of the resulting clear solution through a 0.2 μm PTFE (Fisher Scientific, Leicestershire, UK). This nanogel solution was then diluted to a concentration of 0.1 mg/mL with filtered PBS (0.2 μm PTFE) prior to analysis and loaded in a disposable cuvette (Fisher Scientific, Leicestershire, UK, catalogue no. 15520814). To avoid contamination from airborne dust, all samples were filtered under a fume hood, and cuvettes were flushed with air immediately before samples were added. All measurements were carried out in triplicate using the backscatter (173°) angle mode, allowing 10 min for sample temperature to equilibrate prior to each measurement. All nanogels were analyzed at 20 °C with exception of NG2_20XL_, which was analyzed at 15 °C due to its lower VPTT and to ensure that DLS analysis is carried out at least 10 °C below this value.

For protein corona analysis, stock solutions of Lyso (1 mg/mL) were obtained by dissolving the powders in PBS (10 mM, pH 7.4) without agitation. The clear stock solutions were then filtered through a 0.2 PES filter and diluted to 1 mg/mL by using either filtered PBS (control samples) or nanogel stock solutions (protein corona analysis). To avoid contamination from airborne dust, all samples were filtered under a fume hood, and cuvettes were flushed with air immediately before samples were added. *D*_h_ of each sample was collected (triplicate, backscatter angle mode) at temperatures of 20, 22, 24, 26, 28, 30, 32, 34, 36, 38, 40, 42, and 45 °C allowing 10 min equilibration time at every change in temperature. Once the temperature reached 45 °C, *D*_h_ of the samples was measured while cooling to 20 °C using the same method and at the same temperature intervals.

### Characterization of Nanogels via Z-Potential Analysis

Z-potential analysis of nanogels was conducted using a Zetasizer Nano Ultra operated with ZS Xplorer (version 1.5.0.163) (Malvern Instruments Ltd., UK). Nanogel stock colloidal solutions (1 mg/mL) were obtained by dissolving the dry nanogel powder in phosphate buffer (PB; 10 mM, pH 7.4), followed by sonication for 10 min and filtration of the resulting clear solution through 0.2 μm PTFE into a disposable folded capillary cell (1080, Malvern Instruments Ltd., Malvern, UK). To avoid contamination from airborne dust, all samples were filtered under a fume hood, and the disposable folded capillary cell was flushed with air and rinsed with filtered PB immediately before samples were added. All nanogels were analyzed at 20 °C with exception of NG2_20XL_, which was analyzed at 15 °C due to its lower VPTT and to ensure that DLS analysis is carried out at least 10 °C below this value.

### VPTT Measurements and Reversibility Studies via UV–Vis Spectroscopy

VPTT measurements were carried out via a turbidimetry assay using a Cary 100 UV–vis spectrophotometer (Agilent, Cheadle, UK) equipped with an Agilent Temperature Controller (Agilent, Cheadle, UK). To measure VPTT, nanogel stock colloidal solutions (1 mg/mL) were obtained by dissolving the dry nanogel powder in PBS (10 mM, pH 7.4), followed by sonication for 10 min and filtration of the resulting clear solution through a 0.2 μm PTFE. This nanogel solution was then diluted with pure PBS to a concentration of 0.1 mg/mL, and 800 mL was transferred to a quartz UV–vis (type 104-10-40, Hellma, Essex, UK) cuvette for analysis.

For protein corona analysis, stock solutions for BSA (5.2 mg/mL) and Lyso (1.1 mg/mL) were obtained by dissolving the powders in PBS (10 mM, pH 7.4) without agitation. The clear stock solutions were then filtered through a 0.2 PES filter and diluted to the required concentration using either filtered PBS (control samples) or nanogel stock solutions (protein corona analysis). A blank solution (pure PBS) and a solution containing only the nanogels were employed in each separate replicate of the protein corona study as controls.

All VPTT measurements were carried out by monitoring the absorbance at 500 nm of the samples (pure nanogels solutions, pure proteins solutions, nanogel-protein mixtures, and pure PBS as the blank) as a function of temperature in a range between 15 and 80 °C (depending on the specific nanogel formulation). The heating rate was kept at 0.2 °C/min for all of the analysis. Absorbance data for each sample were blank corrected and converted in transmittance (%) using eq 3
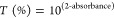
3To obtain the VPTT, the plotted transmittance-temperature data were fitted to a sigmoidal fit (Boltzmann) using eq 4 as given by OriginPro2019 software
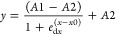
4where *A*1 and *A*2 are the initial and final values of transmittance and d*x* is the change in temperature. The VPTT corresponds to the inflection point of the curve, which is given by Origin software as the parameter *x*_0_. To ensure an accurate estimation of the VPTT, only fitted curves with *R*^2^ > 0.99 were considered.

Changes in VPTT of the nanogels as a function of Lyso concentration were obtained by subtracting the values of VPTT of each NG-Lyso mixture from the values obtained from the relative control sample (nanogels alone).

First derivative data were calculated from the transmittance-temperature data using eq 5
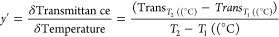
5where the nominator shows the change in transmittance of the sample upon heating from *T*_1_ (°C) to *T*_2_ (°C) and the denominator shows the change in temperature.

In the case of the study of the reversibility of the thermoresponsive transition, samples containing nanogels, Lyso, nanogel-Lyso mixtures, or a blank were first heated from 20 to 45 °C at a rate of 0.2 °C/min. Upon reaching 45 °C, the samples were cooled to 20 °C at the same rate. The absorbance of the samples was monitored at 500 nm throughout both the heating–cooling ramp, blank corrected, and finally converted in transmittance using eq 3. The reversibility of the transition was quantified by comparing the value of transmittance at the end of the cooling ramp for the NG-Lyso mixtures to the value obtained for the relative control sample (nanogels alone).

### Circular Dichroism

CD spectra were obtained with a Chirascan spectrometer (Applied Photophysics, Ltd. Leatherhead UK) using a 1 mm quartz cell (110-4-40 Hellma Analytics). The CD spectra of nanogels, proteins, and their mixtures in PBS (10 mM, pH 7.4) were recorded from 190 to 260 nm (bandwidth 1 nm, time-per-point 1 s) at 20 °C. Data analysis was performed with Chirascan Viewer software. Typically, three scans were acquired and averaged. The resulting average spectrum was subtracted from the contribution of PBS (and nanogels when appropriate), and all spectra were zeroed at 260 nm.

## Results and Discussion

### Synthesis and Characterization of Thermoresponsive Nanogels

**Figure 1:**
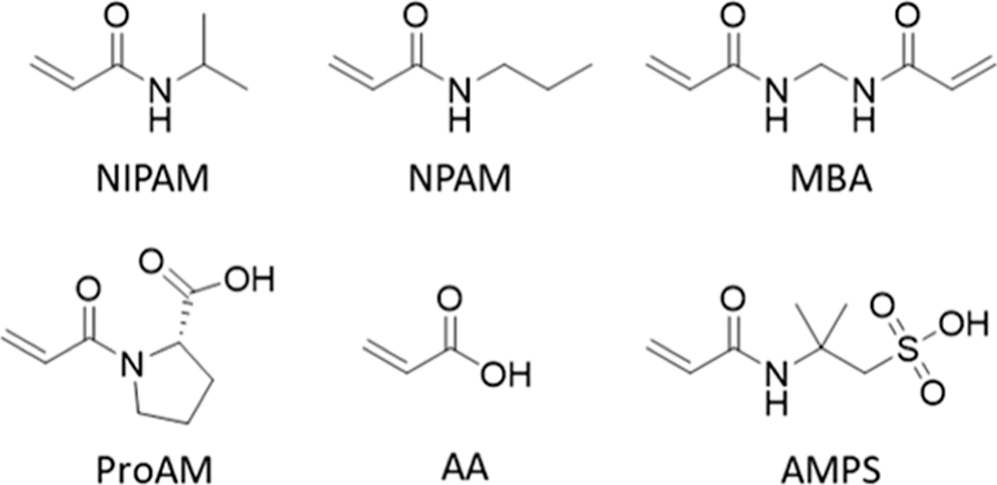
Chemical structure of monomers employed in this study.

Four nanogels (Table 1) were synthesized using HDRP, a method that allows us to obtain nanogels with controllable size and polydispersity, without the requirement of a surfactant.^45,46^ NIPAM and NPAM were chosen as thermoresponsive functional monomers for this study (Figure 1).

**Table 1:** Chemical Composition, Monomer Conversions (^1^H NMR), and Chemical Yields for Neutral and Negatively Charged Nanogels Based on NIPAM or NPAM as Backbone Monomers^a^

	feed composition (molar %)			mon. conversion (%)								
NG	NIPAM	NPAM	ProAM	backbone	MBA	ProAM	Tot	yield (%)	*D*_h_ (nm)	PDI	Z-potential (mV)	VPTT (°C)
NG1_20XL_	80			84	96		92	74	12.5 ± 1.6	0.24	N/A	40.3 ± 0.2
NG2_20XL_		80		90	98	>99	92	75	11.1 ± 0.3	0.25	N/A	28.3 ± 0.7
NG3_20XL_	77.5		2.5	87	97		90	70	7.9 ± 0.6	0.56	–9.1 ± 1.6	54.5 ± 0.4
NG4_20XL_		77.5	2.5	86	97	>99	90	75	8.3 ± 0.5	0.31	–10.8 ± 1.1	44.8 ± 0.4

a All nanogels were synthesized with a fixed amount of MBA equals to 20 molar %. For all the formulations, AIBN was 1% of the total moles of double bonds in the mixture, while the C_M_ was kept constant at 2% (w/w). The hydrodynamic diameter (*D*_h_) by number distribution was obtained by DLS measurements on nanogel suspensions at 0.1 mg/mL in PBS (10 mM, pH 7.4) at 20 °C. NG2_20XL_ was analyzed at 15 °C to ensure adequate distance from the value of VPTT. Z-potential measurements were conducted on NG3_20XL_ and NG4_20XL_ solutions (1 mg/mL) in PB (10 mM, pH 7.4) at 20 °C

NIPAM is a common monomer used for the synthesis of thermoresponsive polymers^47,48^ and nanogels^49^ given its good water solubility and its lower critical solubility temperature (LCST) of 32 °C, which is close to body temperature. NPAM, a structural isomer of NIPAM, was chosen because of its lower LCST (23 °C), which allows us to obtain covalently cross-linked nanogels with VPTT close to physiological values, when combined with negatively charged monomers, as we previously reported.^9,21,45^ Initially, the MBA content was kept at 20 molar %, to obtain a more rigid matrix, suitable for drug delivery applications.^35^ Negatively charged nanogels were obtained by incorporating 2.5 molar % of ProAM, previously employed by our group^21^ to obtain nanogels with dual thermo- and pH-responsive properties. The incorporation of positively charged comonomers with pH-responsive behavior, such as *tert*-butylaminoethyl-methacrylate, was not evaluated in this work due to the observed toxicity of the resulting nanogels in vivo.^38^ All nanogels were synthesized with high total monomer conversions (>90% as determined by ^1^H NMR, Figure S1) and chemical yields (>70%) (Table 1), providing evidence of consistency between the initial formulation and the composition of the isolated polymers. Higher monomer conversions compared to the chemical yields result from the loss of low-molecular-weight chains during the purification step via dialysis (molecular-weight cutoff 3.5 kDa), which also ensures the elimination of any residual monomers that could cause cytotoxicity.

The characterization of particle size was carried out using DLS, which allows us to evaluate the behavior of the swollen nanogels in their colloidal state, as opposed to the dry state using transmission electron microscopy. The *D*_h_ of the nanogels was found to be around 10 nm by DLS (number distribution, Figure S2), while Z-potential analysis carried out on NG3_20XL_ and NG4_20XL_ showed negative charges of −9.1 ± 1.6 mV and −10.8 ± 1.1 mV, respectively, confirming the incorporation of the negatively charged ProAM comonomer, with no significant impact on the nanogels’ size. The similarity in size of the isolated nanogels was deemed sufficient to allow further studies, focusing on the effects of protein corona formation on the thermoresponsive behavior.

### Effect of Proteins on the VPTT of Neutral and Charged Nanogels

**Figure 2:**
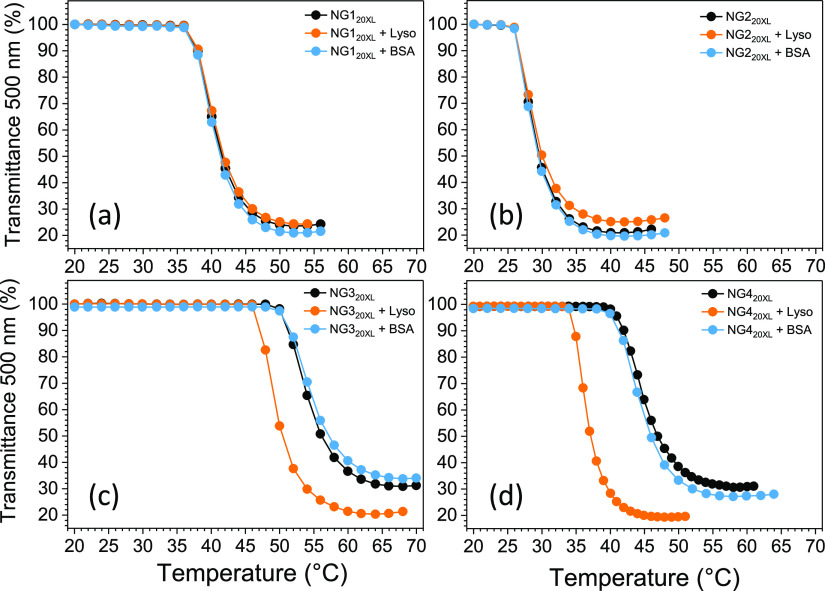
Thermoresponsive behavior of nanogels (0.1 mg/mL, black) in the presence of either Lyso (1 mg/mL, orange) or BSA (4.7 mg/mL, blue). (a) NG1_20XL_ (NIPAM neutral), (b) NG2_20XL_ (NPAM neutral), (c) NG3_20XL_ (NIPAM negative), and (d) NG4_20XL_ (NPAM negative).

The VPTT of the nanogels, at 0.1 mg/mL, was first measured in PBS solutions (10 mM, pH 7.4) by using UV–vis spectroscopy (transmittance change at 500 nm). VPTT values were obtained as the inflection point of the sigmoidal fit (Boltzmann) (Figure 2a–d). The neutral nanogel NG1_20XL_ (NIPAM) presented a VPTT of 40.3 ± 0.2 °C, higher than the 28.3 ± 0.7 °C found for NG2_20XL_ (NPAM), due to the higher hydrophobicity of the linear substituent of NPAM compared to the branched one of NIPAM.^21,50^ Negatively charged nanogels NG3_20XL_ (NIPAM) and NG4_20XL_ (NPAM) showed values of VPTT of 54.5 ± 0.4 and 44.8 ± 0.4 °C, respectively, higher than their neutral counterparts (+14.2 and +16.5 °C for NG3_20XL_ and NG4_20XL_, respectively). The presence of the carboxylate groups from the ProAM units results in more hydrophilic polymers, leading to higher VPTT values,^51,52^ also allowing some tailoring of the thermoresponsive properties. The difference in the values of VPTT is fully justified by the differences in each formulation.

To evaluate the effect of protein interactions on VPTT, Lyso (pI 11)^53^ and BSA (pI 4.8),^44^ previously used as model proteins by other groups, were employed in PBS.^54−56^ The positive charge of Lyso and its high concentration in saliva,^40^ tears,^41^ and airways mucus,^42^ make it relevant to study its impact on thermoresponsive behavior, while BSA with its negative surface charge was chosen for comparison. Both proteins were used at the same concentration (71.2 μM) to ensure good solubility in PBS. UV–vis spectroscopy and DLS (Figure S3) were used to confirm that both proteins remain colloidally stable at these concentrations, up to temperatures of 65 °C. Similar experiments were carried out using BSA as a negative control as this protein has an overall negative charge, using both neutral and negatively charged polymers; as expected no significant changes in VPTT values were observed, confirming the absence of interactions (Figure 2). A similar result was found when Lyso, positively charged, was added to the neutral NG1_20XL_ (NIPAM) and NG2_20XL_ (NPAM); the small size of these nanogels may have contributed to limiting the influence of hydrophobic interactions with proteins.^57−59^ The addition of Lyso to the negatively charged nanogels NG3_20XL_ (NIPAM) and NG4_20XL_ (NPAM) led to VPTT of 49.9 ± 0.2 and 36.8 ± 0.3 °C, respectively, which are significantly lower than the values of the nanogels alone (−4.6 ± 0.3 °C and −7.9 ± 0.2 °C). The differences in VPTT observed for NG3_20XL_ (NIPAM) and NG4_20XL_ (NPAM) suggest that the NG-Lyso complexes present a lower hydrophilicity compared to the nanogels, likely due to the electrostatic nature of the interactions between the positively charged groups on Lyso and the carboxylate units on the polymers. Indeed, when the thermoresponsive behavior of nanogels alone was evaluated at pH 4.2 (10 mM acetate buffer saline) (table S1) a similar drop in VPTT values was observed due to the partial protonation of ProAM in the acidic environment and the resulting decrease in hydrophilicity of the polymer (pH-responsive property). NG4_20XL_ (NPAM) showed a larger drop in VPTT compared to NG3_20XL_ (NIPAM) upon interaction with Lyso due to the different degrees of interaction with the protein, given the differences in the structure of the side chain of the backbone monomers. Given the widely reported role of the hydrophobic effect in promoting the formation of protein corona, we hypothesize that the linear propyl group of NPAM, which presents a larger surface area compared to the branched isopropyl group in NIPAM, may offer additional Van Der Waals interactions with Lyso, leading to larger effects on the VPTT of the nanogel.

### Impact of the Charged Comonomer and Cross-Linker Content Structure on VPTT of NPAM Nanogels

The biologically relevant VPTT value of NG4_20XL_ (NPAM) directed the focus of the work toward further evaluating the impact of complex formation with Lyso on the VPTT of negatively charged NPAM-based nanogels. To further expand the scope of our initial finding, and given that the chemical structure of the charged moieties on nanoparticle’s surfaces has been shown to impact protein corona formation,^60−62^ two additional functional monomers were chosen to be incorporated into the formulation, AA and AMPS. These units have been previously used to introduce negative surface charges, pH-responsive behavior, and to also tailor the VPTT of nanogels.^63^ Additionally, changes in the concentration of MBA between 2.5 and 20 molar % allowed us to evaluate the role of matrix rigidity on the interaction with the protein; previous results showed how nanogels with a lower degree of XL present a more flexible matrix,^64^ that undergoes more extensive conformational changes in response to temperature^65,66^ and upon adsorption to hydrophobic interfaces.^67^ Particles’ rigidity has also been shown recently to impact silica nanocapsules^68^ and iron oxide nanoparticles,^69^ while for nanogels, the cross-linker effect has been reported in terms of changes in surface charge density.^70^

Nanogels were obtained with good total monomer conversions (>85%, Figure S1) and yields (>74%) (Table 2). The *D*_h_ for the nanogels, measured in PBS were found to be consistently in the range 6–10 nm, with polydispersity < 0.56 and z-potential values ranging between −8 and −12 mV. The similarities in size and surface charge provide evidence that the incorporation of the negatively charged functional monomers resulted in particles with comparable morphology.

**Table 2:** Chemical Composition of Negatively Charged Nanogels Incorporating 2.5 Molar % of Either ProAM, AA, or AMPS, and with Varying Amounts of XL^a^

NG_%XL_	NPAM (molar %)	MBA (molar %)	neg. charged monomer (molar %)	tot conv. (%)	yield (%)	*D*_h_ (nm)	PDI (nm)	Z-pot (mV)	VPTT (°C)	thermal hysteresis (°C)
NG5_2.5XL_	95	2.5	ProAM (2.5 molar %)	92 ± 1	80 ± 3	7.4 ± 0.5	0.42	–11.7 ± 0.9	44.2 ± 1.2	7.6 ± 0.7
NG6_5XL_	92.5	5		88 ± 1	81 ± 4	8.3 ± 0.8	0.56	–9.6 ± 0.6	43.5 ± 1.1	4.3 ± 0.3
NG7_10XL_	87.5	10		87 ± 6	86 ± 2	8.3 ± 1.7	0.50	–7.7 ± 1.6	43.2 ± 0.8	3.0 ± 0.4
NG4_20XL_	77.5	20		90 ± 1	75 ± 6	8.3 ± 0.5	0.31	–10.8 ± 1.1	44.8 ± 0.4	1.1 ± 0.4
NG8_2.5XL_	95	2.5	AA (2.5 molar %)	92 ± 2	81 ± 2	6.1 ± 0.7	0.30	–9.7 ± 1.3	48.6 ± 1.6	11.0 ± 0.8
NG9_5XL_	92.5	5		92 ± 1	82 ± 5	7.6 ± 0.3	0.29	–9.3 ± 0.1	47.6 ± 0.2	7.0 ± 0.6
NG10_10XL_	87.5	10		93 ± 0	85 ± 7	8.8 ± 1.2	0.51	–8.5 ± 0.6	46.5 ± 0.6	3.9 ± 0.3
NG11_20XL_	77.5	20		94 ± 1	86 ± 13	10.5 ± 3.0	0.29	–8.5 ± 0.8	49.1 ± 0.6	2.7 ± 0.2
NG12_2.5XL_	95	2.5	AMPS (2.5 molar %)	90 ± 0	82 ± 3	7.5 ± 0.6	0.26	–8.9 ± 0.8	35.9 ± 0.5	6.4 ± 0.5
NG13_5XL_	92.5	5		85 ± 9	74 ± 16	6.2 ± 0.4	0.44	–9.2 ± 1.3	35.3 ± 1.1	3.3 ± 0.9
NG14_10XL_	87.5	10		90 ± 1	78 ± 8	6.6 ± 0.4	0.53	–8.5 ± 0.9	35.1 ± 1.1	1.9 ± 0.2
NG15_20XL_	77.5	20		87 ± 2	82 ± 11	8.2 ± 0.4	0.34	–8.4 ± 1.8	36.5 ± 1.5	1.5 ± 0.4

a Total monomer conversions were estimated with ^1^H NMR, while *D*_h_ (by number at 20 °C) and PDI were determined by DLS on nanogel colloidal solutions (0.1 mg/mL) in PBS (10 mM, pH 7.4). VPTT and hysteresis values were measured with UV–vis on the same nanogel solution. Z-potential measurements were conducted on nanogel solutions (1 mg/mL) in PB (10 mM, pH 7.4) at 20 °C

VPTT values were obtained for all the formulations to evaluate the effect of the increased rigidity; the data clearly show that when the cross-linker content was increased nearly nine folds, from 2.5 to 20% MBA, within the same formulation, the nanogels displayed transition temperatures that were similar. However, when the functional monomer was changed, the structure of the negatively charged groups significantly affected the VPTT; ProAM-nanogels showed a mean VPTT value of 43.9 ± 0.4 °C, while the addition of AA and AMPS resulted in nanogels with mean VPTT values of 35.7 ± 0.4 and 47.9 ± 1.1 °C, respectively (Table 2, Figure S4a–l).

Given the high monomer conversions of the functional monomers (table S2) and their expected ionized state at pH 7.4 (p*K*_a__(AMPS)_ = 2.3,^71^ p*K*_a__(AA)_, and p*K*_a__(ProAM)_ both <5^71,72^), the results can be explained by the different hydrophobic character of the charged functional monomer, which is closely related to their chemical structure. AA is highly hydrophilic and leads to polymers with the highest VPTT in this series; AMPS and ProAM both have four carbon atoms in the side group, which makes them more hydrophobic compared to AA, and in fact, their VPTTs are lower than the AA nanogels. In the case of AMPS, the highly branched structure is more hydrophobic and is responsible for the lower VPTT.

When we implemented a heating/cooling cycle and monitored transmittance of the polymer solutions, all nanogels were found to have a fully reversible thermoresponsive behavior (Figure S4). However, the cross-linker content was shown to have an impact on the transition temperature recorded during the cooling phase, leading to a significant hysteresis, especially for the more flexible nanogels (Table 2). As polymer–polymer interactions have been previously identified as factors influencing hysteresis in thermoresponsive polymers,^73^ we explained our results with the more extensive conformational changes of the more flexible nanogels.

### Effect of Lyso Addition on the VPTT of NPAM Nanogels: The Role of Charged Monomer Structure and Cross-Linker Contents

**Figure 3:**
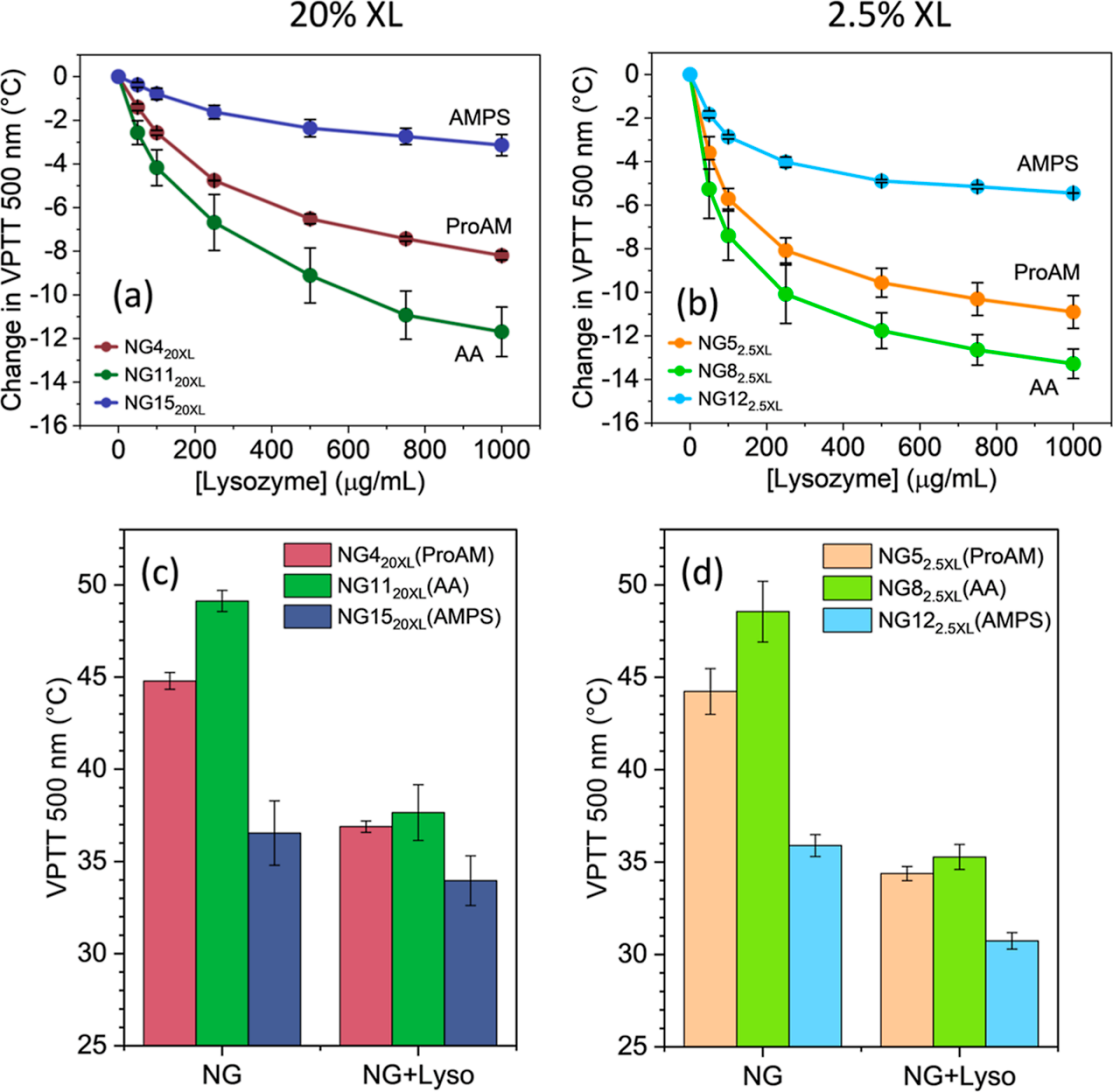
Impact of nanogel-Lyso interactions on VPTT. Change in VPTT of negatively charged nanogels incorporating ProAM (orange), AA (green), and AMPS (blue) with either (a) 20 molar % MBA or (b) 2.5 molar % MBA as a function of Lyso concentration. Average values (*n* ≥ 3) of VPTT for nanogels alone or in the presence of 1 mg/mL Lyso are presented for nanogels with (c) 20 molar % MBA or (d) 2.5 molar % MBA.

The VPTT of nanogels incorporating ProAM, AA, or AMPS and having either 2.5 or 20 mol % XL were measured in PBS (10 mM, pH 7.4) with increasing concentration of Lyso (0–1 mg/mL). For nanogels with 20 molar % XL, the presence of AMPS led to a drop in VPTT of −2.5 ± 0.5 (from 36.5 to 34.0 °C) at the highest protein concentration (Figure 3a), while nanogels containing ProAM and AA led to a decrease in VPTT of −7.9 ± 0.2 °C and −11.5 ± 1.1 °C, respectively (Figure S5 and Table S3 for the complete set of data). This provides further evidence of the importance of negatively charged monomers in driving the formation of NG-Lyso complexes.

Data also suggest a role played by the structure of the charged monomer in influencing the degree of change in VPTT, in the order AMPS > ProAM > AA, which is consistent with their increasing hydrophilicity. This could be complemented by the differences in the steric hindrance of the negatively charged moieties, reducing the binding of Lyso. While AA offers a more accessible binding site for Lyso, the presence of a 2-methylpropyl- substituent in AMPS and the pyrrolidine in ProAM provides higher steric hindrance, limiting the formation of the complex. The more rigid (and thus easily accessed) pyrrolidine group in ProAM compared to that in AMPS may also explain the larger effect observed in the nanogels containing the former.

When the impact of the XL content was evaluated using nanogels with 2.5 mol % MBA, the general trend was retained, revealing the central role of the chemical composition in controlling the effect of protein corona formation on the VPTT of the nanogels. However, data in Figure 3b (Figure S5 and Table S3) also show that complex formation with 2.5 molar % XL nanogels led to a slightly larger drop in VPTT, revealing how the matrix rigidity may also contribute to the final thermoresponsive behavior of the complex; more flexible nanogels may better adapt to the protein structure, leading to more binding sites and larger effects on the polymer’s thermoresponsive behavior.

When the VPTTs for the NG-protein complexes are compared to the values for the nanogels alone, the differences originally due to the chemical structure of the varied functional monomers are considerably reduced. This observation was found to be true for polymers with 2.5 and 20% cross-linker content (Figure 3c,d). The formation of a protein corona may have resulted in a “leveling effect”, attenuating the impact of the chemical composition on the nanogel’s thermoresponsive behavior. This behavior further highlights the importance of introducing protein corona studies when developing thermoresponsive nanomaterials.

These data show that while designing drug delivery systems based on thermoresponsive polymers, potential changes in behavior of the nanomaterial in response to interactions with biomolecules need to be carefully considered.

### Reversibility of the Thermoresponsive Behavior of Polymeric Nanogels in the Presence of Lyso

**Figure 4:**
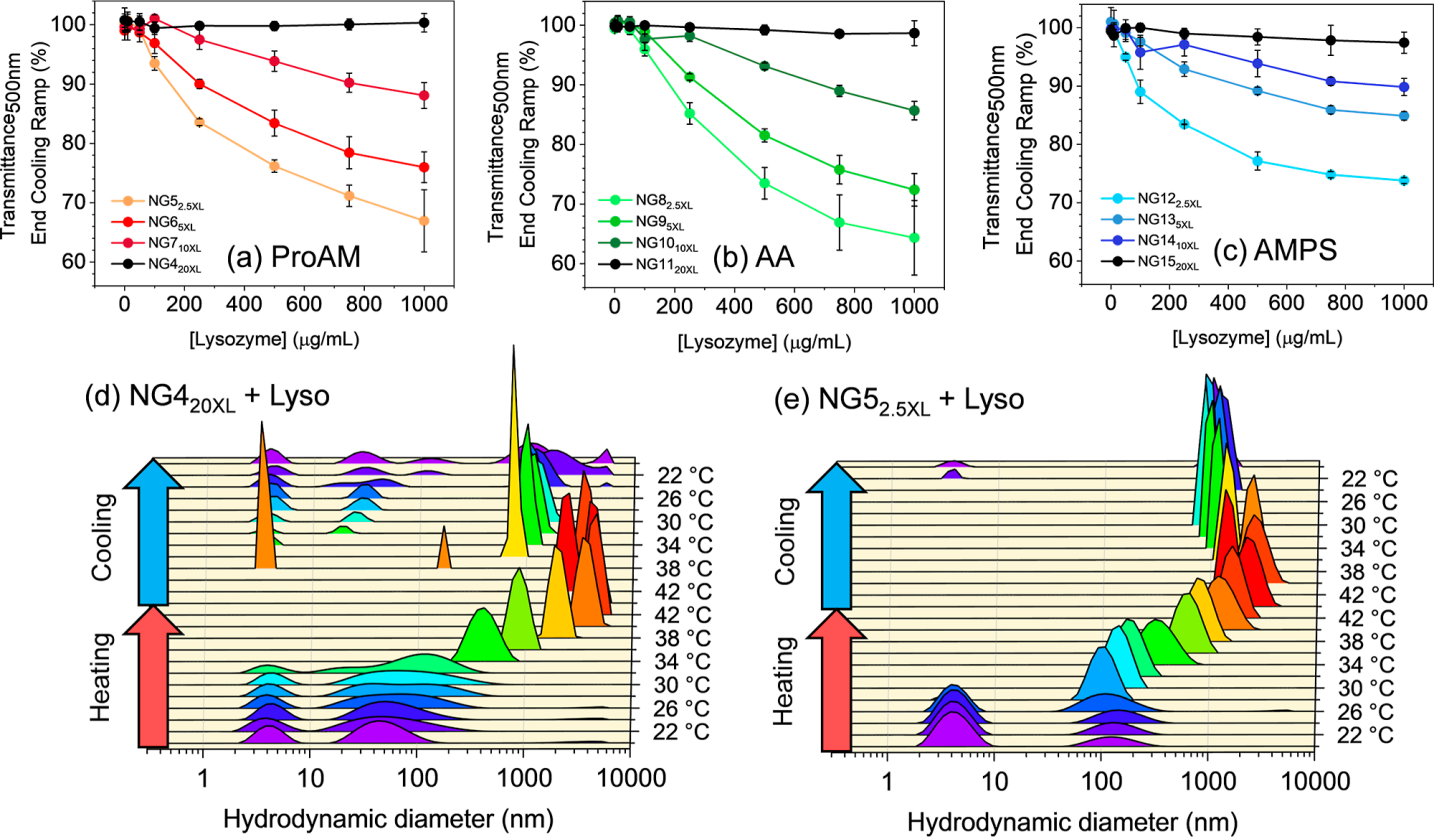
Impact of protein corona formation on the reversibility of the thermoresponsive behavior of nanogels. The transmittance of NG-Lyso mixtures at the end of the heating/cooling cycle for nanogels of varying XL density and incorporating either (a) ProAM, (b) AA, or (c) AMPS as charged comonomers reveal irreversible transitions for more flexible nanogel matrixes. Size analysis by DLS (intensity distribution) of (d) NG4_20XL_ + Lyso and (e) NG5_2.5XL_ + Lyso as a function of temperature confirms irreversible increase in size for the less cross-linked nanogels upon complexation with Lyso.

Given the results obtained for the effect of protein corona formation on the VPTT of nanogels, we then focused on evaluating the impact of complex formation on the reversibility of the nanogels. Nanogel solutions (0.1 mg/mL) with increasing amounts of Lyso (0–1 mg/mL) were heated from 20 to 45 °C (0.2 °C/min), a temperature well below the protein’s denaturation point (Figure S3), and then cooled to 20 °C at the same rate (Figure S6). The transition’s reversibility for each sample was estimated by measuring the transmittance at the end of the heating/cooling cycle and plotting it as a function of protein concentration (Figure 4a–c).

Data show that all nanogels in the absence of Lyso showed 100% transmittance at the end of the experiment, which demonstrated full reversibility under these conditions, as also confirmed by DLS (Figure S7). The addition of Lyso to nanogels with 20 mol % XL led to no significant changes in reversibility (Figure 4a–c). However, a Lyso-concentration-dependent drop in the transmittance of nanogels with XL ≤ 10 mol % at the end of the heating/cooling ramp was observed (Figures 4a–c and S6), suggesting that more flexible nanogels led to the formation of NG-Lyso complexes with higher stability. Interestingly, nanogels with different charged comonomers led to similar results in terms of reversibility of the VPTT transition, which indicates that this parameter does not play a major role in determining the stability of the NG-Lyso complex once the temperature of the solution is lowered.

The correlation between the cross-linker content and thermoreversibility was confirmed by DLS using ProAM-containing NG4_20XL_ and NG5_2.5XL_, Lyso as the protein, under comparable experimental conditions. The results showed that the more flexible polymer NG5_2.5XL_ (Figure 4e) formed large aggregates with Lyso that persisted at the end of the heating–cooling cycle, indicating irreversibility. CD, carried out on the nanogel-protein solutions at the start and at the end of the cycle, showed no evidence of denaturation (Figure S8); this strongly suggests that the data obtained by UV–vis and DLS were not the result of protein denaturation-induced aggregation. These data together indicate that reswelling of the nanogels is hindered by protein corona formation, possibly due to favorable NG-Lyso electrostatic interactions, preventing the polymer from fully reverting its conformational rearrangement. However, when the XL content reaches 20 molar %, the more rigid matrix undergoes less structural rearrangement, allowing the complex to fully resuspend at the end of the heating/cooling cycle. This was further confirmed when observing that values of thermal hysteresis for the 20 molar % cross-linked nanogels were not affected upon complexation with Lyso (table S4). Evaluation of thermal hysteresis for NG-Lyso complexes for nanogels with XL < 20 molar % was not possible due to poor sigmoidal fitting of the cooling ramps (figure S6). Collectively, both the VPTT and reversibility data indicate that once a protein corona is formed on thermoresponsive nanogels, both the chemical composition and morphology of the nanogels may play a role in shaping the thermoresponsive properties of the resulting complex. The surface chemistry of the nanogel, especially with charged groups, has a strong influence on the hydrophilicity of the matrix, therefore impacting the VPTT profile of the complex; the rigidity of the nanogel’s matrix however has been shown to play a role in influencing the reversibility of the transition, an effect that potentially could affect the behavior of the particles in vivo.

## Conclusions

Fine tuning the value of VPTT for thermoresponsive nanoparticles is key to their applications in nanomedicine and requires a deeper understanding of the impact of potential interactions with biomolecules, especially in the case of intranasal and ocular delivery routes. We evaluated the effect of protein corona formation on the thermoresponsive properties of acrylamide-based nanogels, either neutral or negatively charged, and with different rigidity, as a result of varied cross-linker content in the formulations. The positively charged Lyso was chosen, given its relevance for ocular and intranasal drug delivery, while BSA was used as a negative control. The results demonstrate the role that electrostatic interactions between Lyso and NGs have on the VPTT values, with neutral nanogels showing no significant variation, while the negatively charged ones display a drop of 4 or 8 °C, depending on the backbone monomer. The drop in VPTT upon complexation with Lyso was confirmed for three different negatively charged monomers (AMPS, ProAM, and AA); however, a strong dependence on the hydrophilic nature of the negatively charged monomer used in the formulation was observed, e.g., 2–3 °C drop for the more hydrophobic AMPS vs 11–14 °C for the more hydrophilic AA. The role of the cross-linker content on the VPTT values was also studied for all the negatively charged formulations, showing a limited but statistically significant effect. In addition, the reversibility of the temperature-dependent transition of nanogels in the presence of Lyso was studied, given the importance of this property for drug delivery systems. Interestingly, in this case, the cross-linker content was found to play a fundamental role, with the more rigid matrix (20 mol % MBA) showing full reversibility, while the more flexible nanogels (<10 mol % MBA) led to the formation of irreversible aggregates. Our results provide evidence that the formation of protein corona alters the thermoresponsive behavior of the nanogels, both in terms of VPTT values and reversibility of the transition, with potential impact on the release of small drugs and biopharmaceuticals. The study provides insights on how surface chemistry and rigidity of the nanogels may be tailored to control the formation of the protein corona on nanogels and the thermoresponsive behavior of the resulting complexes. Moreover, the data in this work highlight that the formation of a protein corona may act as an additional external stimulus that could be coupled with the thermoresponsive behavior of the nanogels to obtain a protein corona-responsive materials.

## Supporting Information Available

Nanogel characterization data (^1^H NMR monomer conversion, DLS, and VPTT analysis), protein temperature stability studies, and complete set of VPTT graphs showing the impact of Lyso on thermoresponsive behavior of nanogels (both heating and cooling ramps) (PDF)
