# Supplementary material for: Impact of Protein Corona Formation on the Thermoresponsive Behavior of Acrylamide-Based Nanogels
Source: Biomacromolecules. 2024 Jan 19;25(2):1340–50. doi: 10.1021/acs.biomac.3c01405 (PMC10865348; doi:10.1021/acs.biomac.3c01405)
Supplement: Supplementary file 1 — bm3c01405_si_001.pdf [file bm3c01405_si_001.pdf]

SUPPLEMENTARY INFORMATION

Impact of protein corona formation on the thermoresponsive behaviour of acrylamide-based nanogels

Federico Traldi and Marina Resmini *

Department of Chemistry, SPCS, Queen Mary University of London, London E1 4NS, UK; f.traldi@qmul.ac.uk (F.T.)
* Correspondence: m.resmini@qmul.ac.uk (M.R.)

Figure S1. ^{1}H-NMR spectra for NG1-15 before (black, t = 0 h) and after polymerisation (red, t = 24 h). Peaks integrated to obtain monomer conversion data are indicated with arrows and associated with the chemical structure of the relative monomer, crosslinker or internal standard.

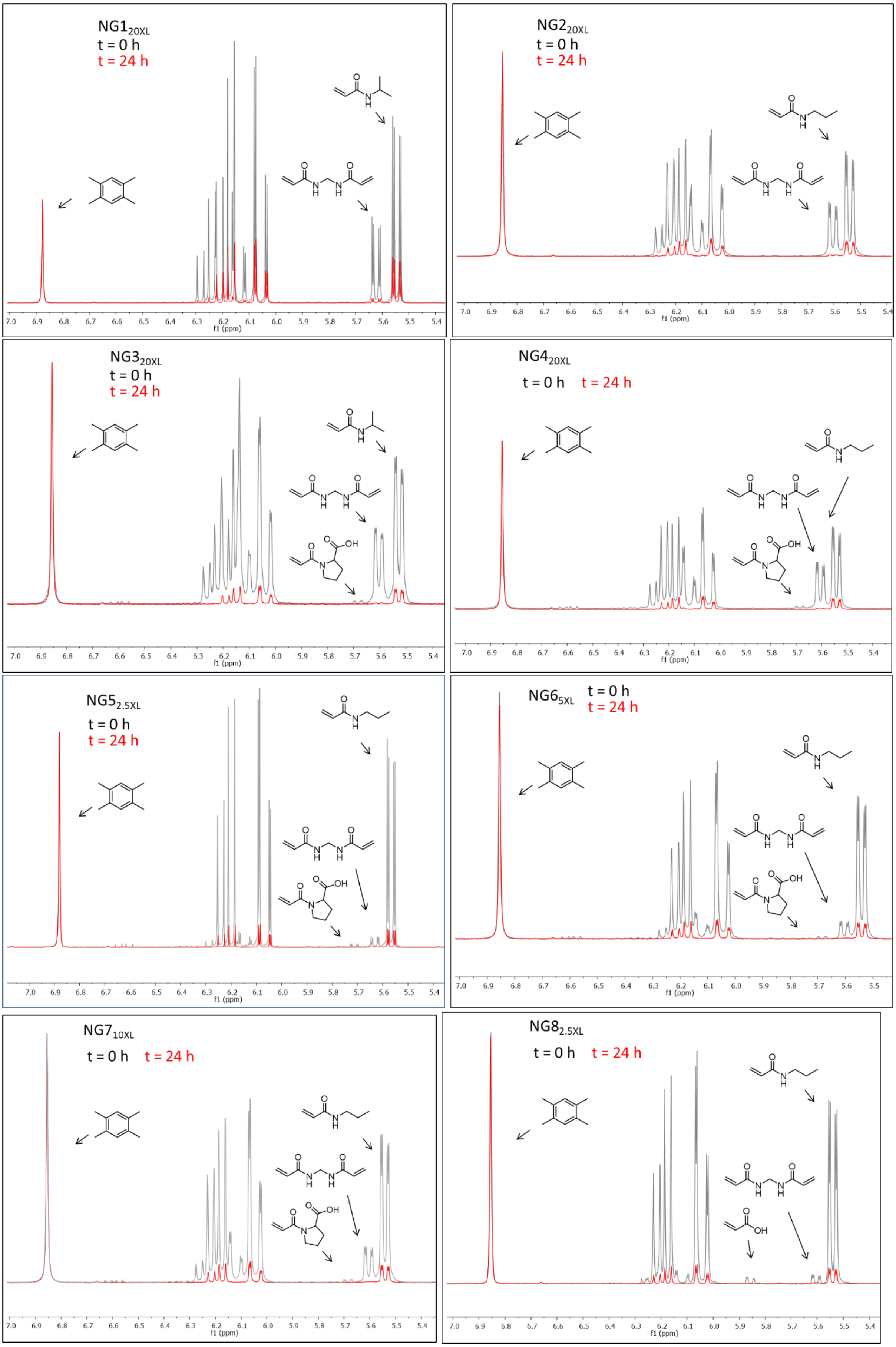

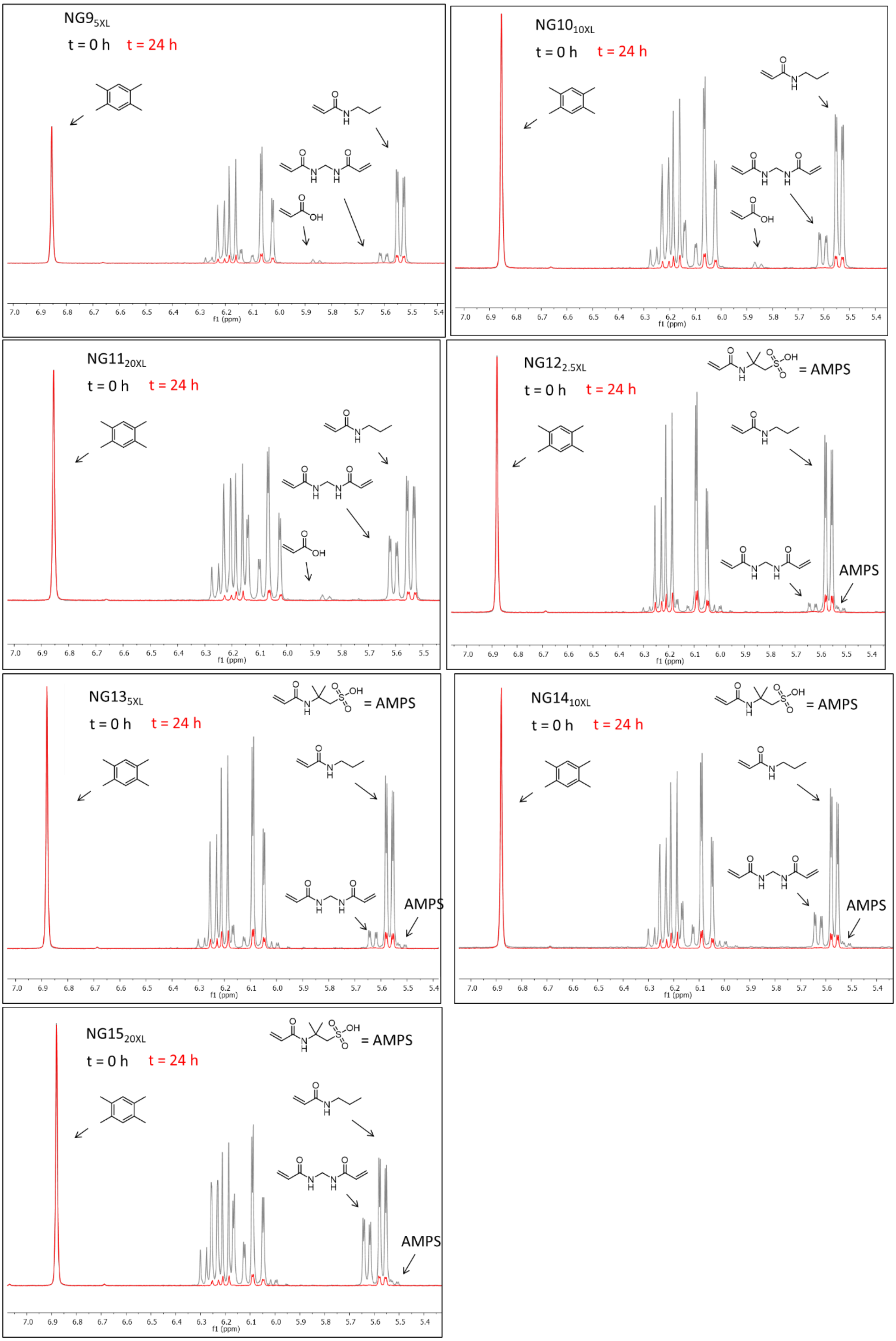
NG9$_{5XL}$
t = 0 h
t = 24 h
NG10$_{10XL}$
t = 0 h
t = 24 h
NG11$_{20XL}$
t = 0 h
t = 24 h
NG12$_{2.5XL}$
t = 0 h
t = 24 h
= AMPS
AMPS
NG13$_{5XL}$
t = 0 h
t = 24 h
= AMPS
AMPS
NG14$_{10XL}$
t = 0 h
t = 24 h
= AMPS
AMPS
NG15$_{20XL}$
t = 0 h
t = 24 h
= AMPS
AMPS
f1 (ppm)

Figure S2. Dynamic light scattering (DLS) data of nanogels synthesised in this work. Data (triplicate measurements) by intensity (orange), volume (black), and number (blue) distribution are presented along with the correlation function.

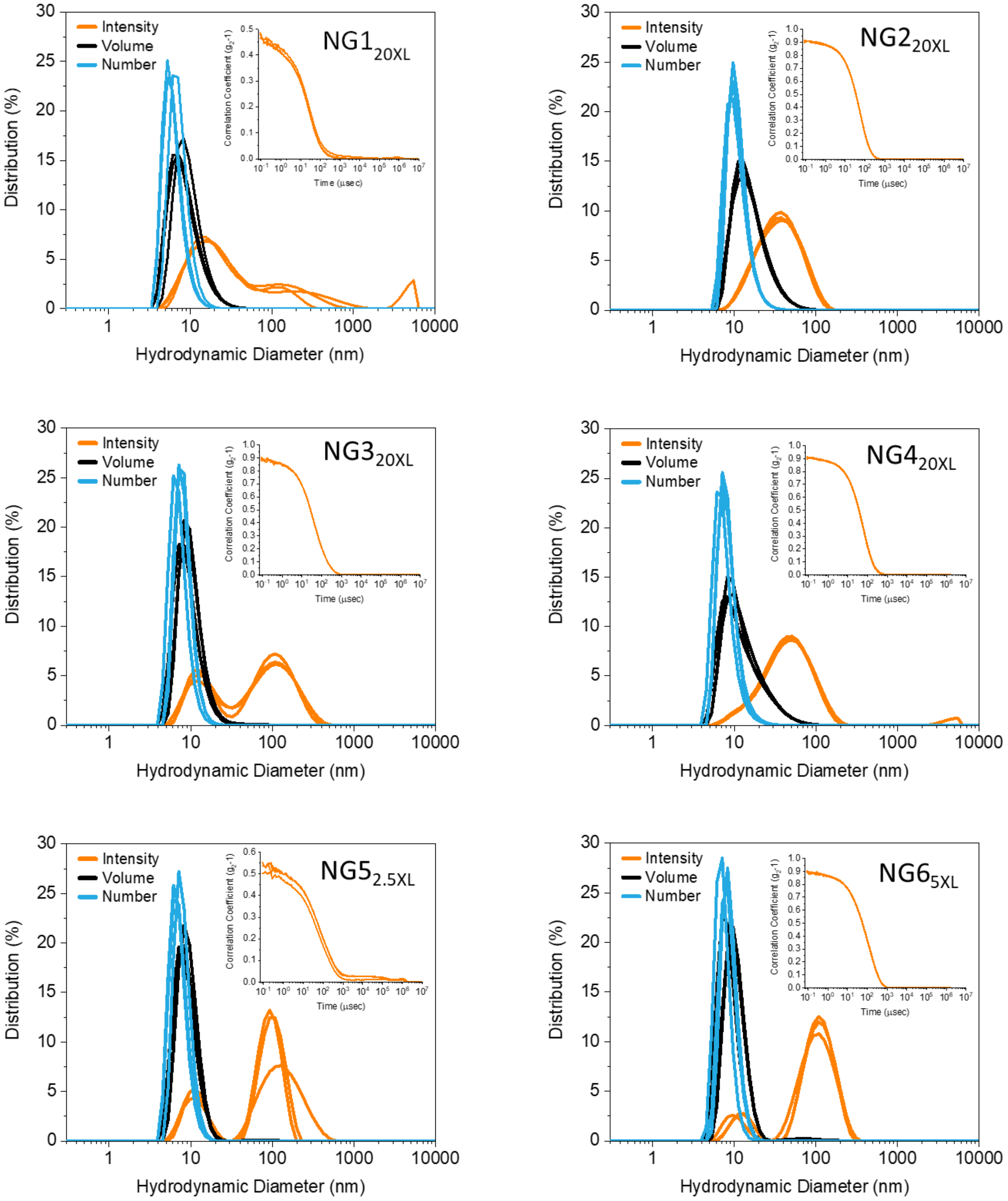

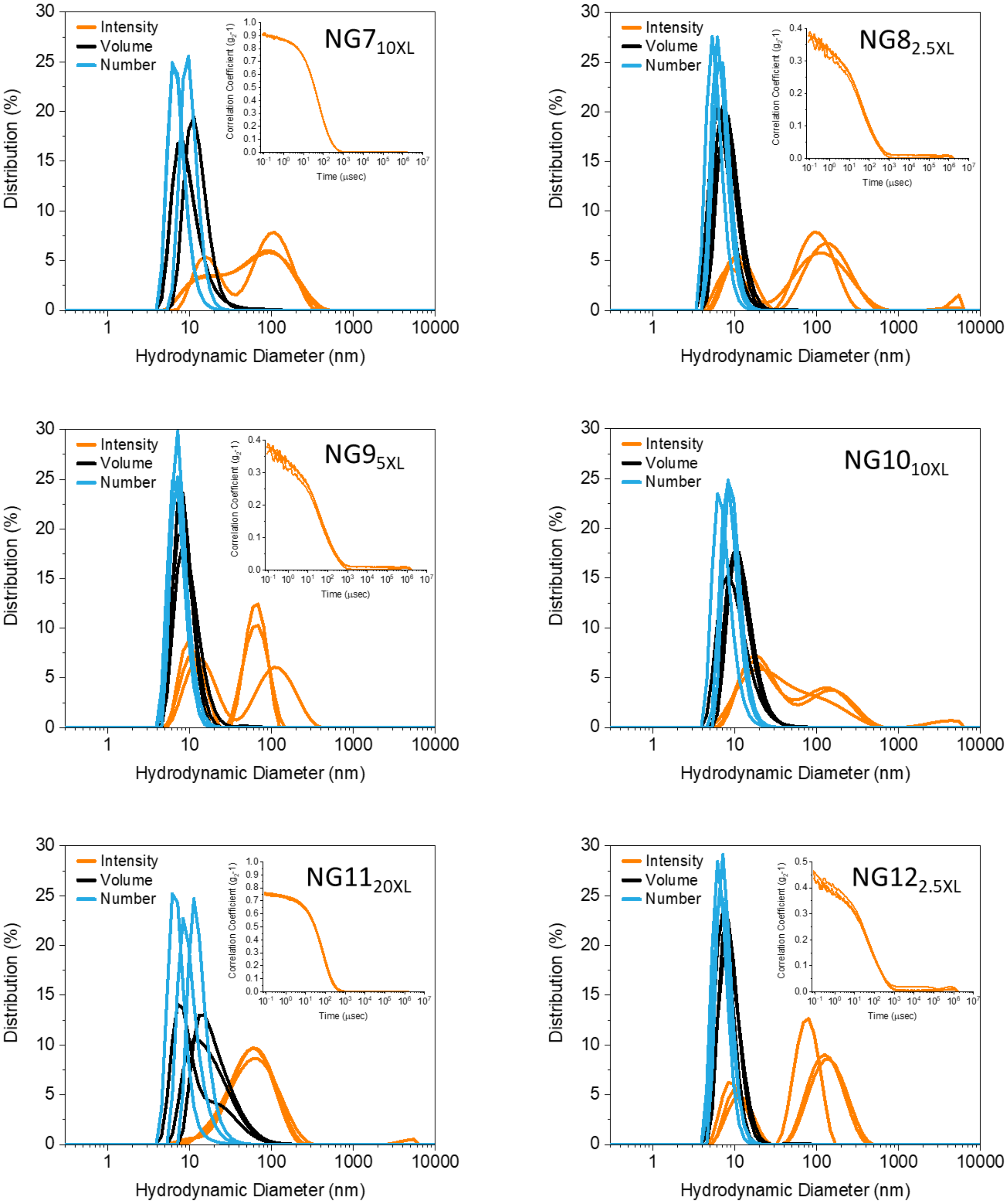
NG7$_{10XL}$
NG8$_{2.5XL}$
NG9$_{5XL}$
NG10$_{10XL}$
NG11$_{20XL}$
NG12$_{2.5XL}$
Intensity
Volume
Number
Distribution (%)
Hydrodynamic Diameter (nm)
Correlation Coefficient (g_2-1)
Time (μsec)

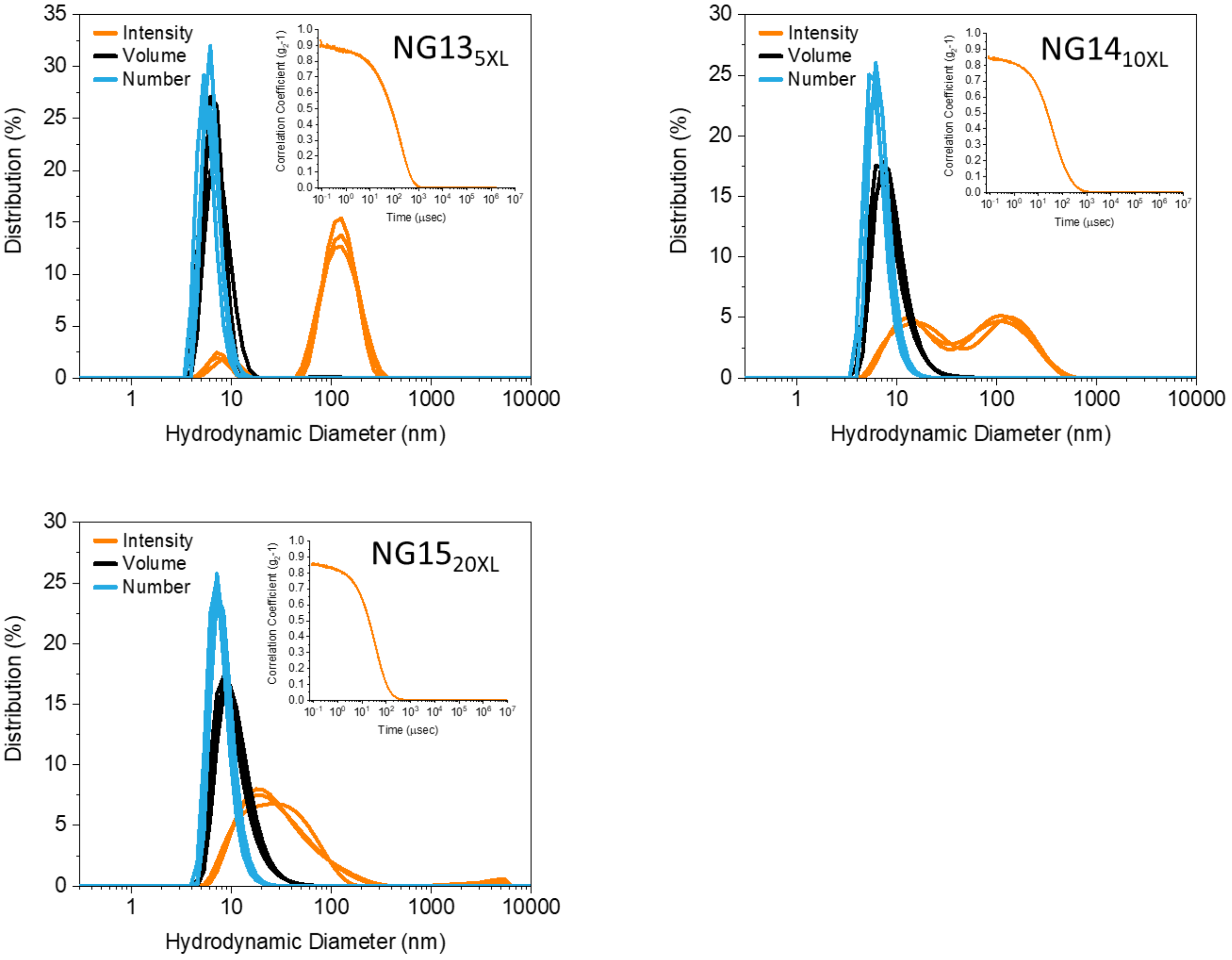
NG13$_{5XL}$
NG14$_{10XL}$
NG15$_{20XL}$
Intensity
Volume
Number
Distribution (%)
Hydrodynamic Diameter (nm)
Correlation Coefficient (g_2-1)
Time (μsec)

Figure S3. Studies of colloidal stability of BSA (4.7 mg/mL) and Lyso (1 mg/mL) in PBS (10 mM, pH 7.4) between 20 – 70 °C via: a) UV-Vis spectroscopy and b-c) intensity based DLS (b and c for BSA and Lyso rispectively).

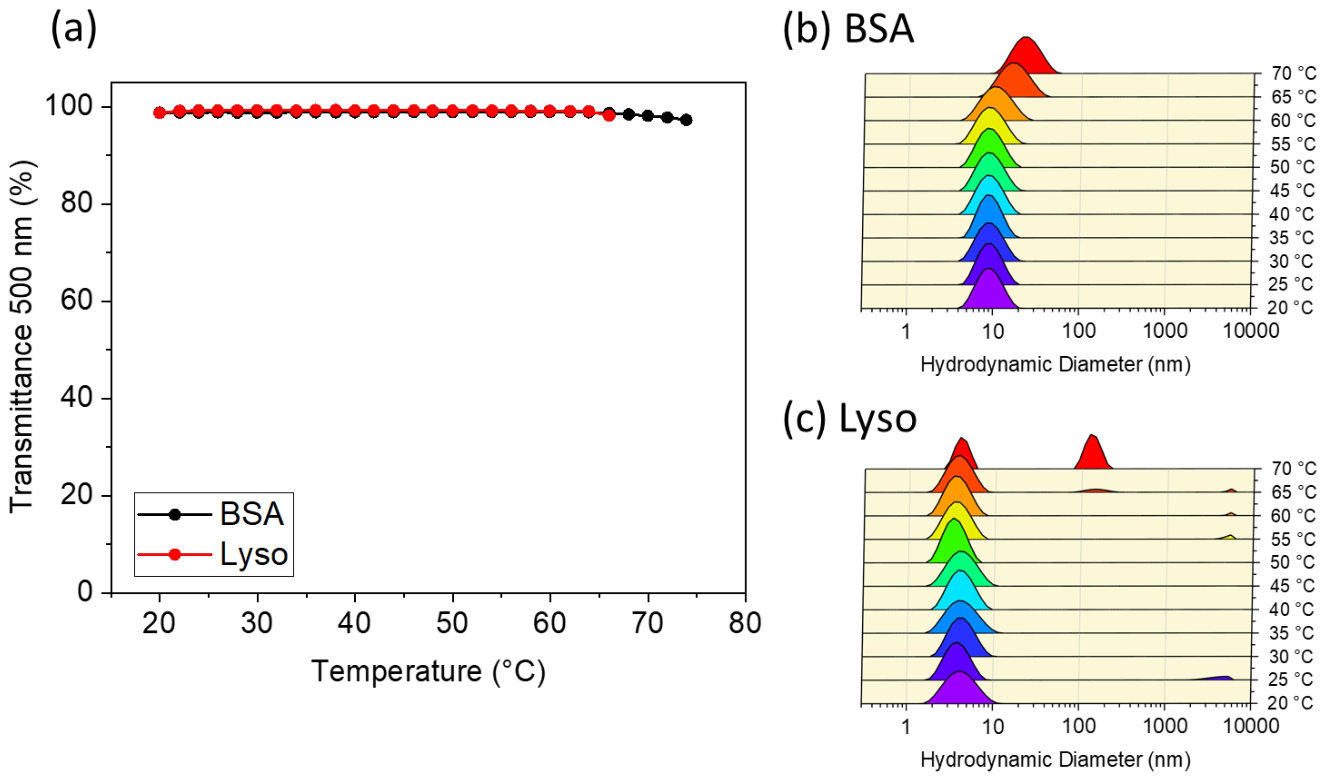

Table S1. Volume phase transition temperature (VPTT) values of neutral $NG1_{20XL}$ (NIPAM), neutral $NG2_{20XL}$ (NPAM), negatively charged $NG3_{20XL}$ (NIPAM) and negatively charged $NG4_{20XL}$ (NPAM) at pH 7.4 or 4.2. All nanogels were synthesised with fixed a amount of MBA (crosslinker, XL) equals to 20 molar%. Phosphate buffer saline (PBS, 10 mM) was employed to obtain pH 7.4, while acetate buffer saline (10 mM) was employed to obtain pH 4.2.

Nanogel Code	Feed Monomer Composition (molar%)			VPTT (°C)	
	NIPAM	NPAM	ProAM	pH 7.4	pH 4.2
$NG1_{20XL}$	80	-	-	40.3	40.9
$NG2_{20XL}$	-	80	-	28.3	28.9
$NG3_{20XL}$	77.5	-	2.5	54.5	44.3
$NG4_{20XL}$	-	77.5	2.5	44.6	33.2

Figure S4. Analysis of the thermoresponsive behaviour of negatively charged nanogels incorporating either ProAM, AA, or AMPS (from left to right), and with increasing molar% of XL (top to bottom). Transmittance of the samples was monitored at 500 nm while heating (orange) and then cooling (blue) the sample at a rate of 0.2 °C/min. The raw data (dots) were fitted (line) using a sigmoidal curve fit (Boltzmann fit, OriginPro 2019). The inflection points of the curves were obtained from the fitting and taken as the VPTT.

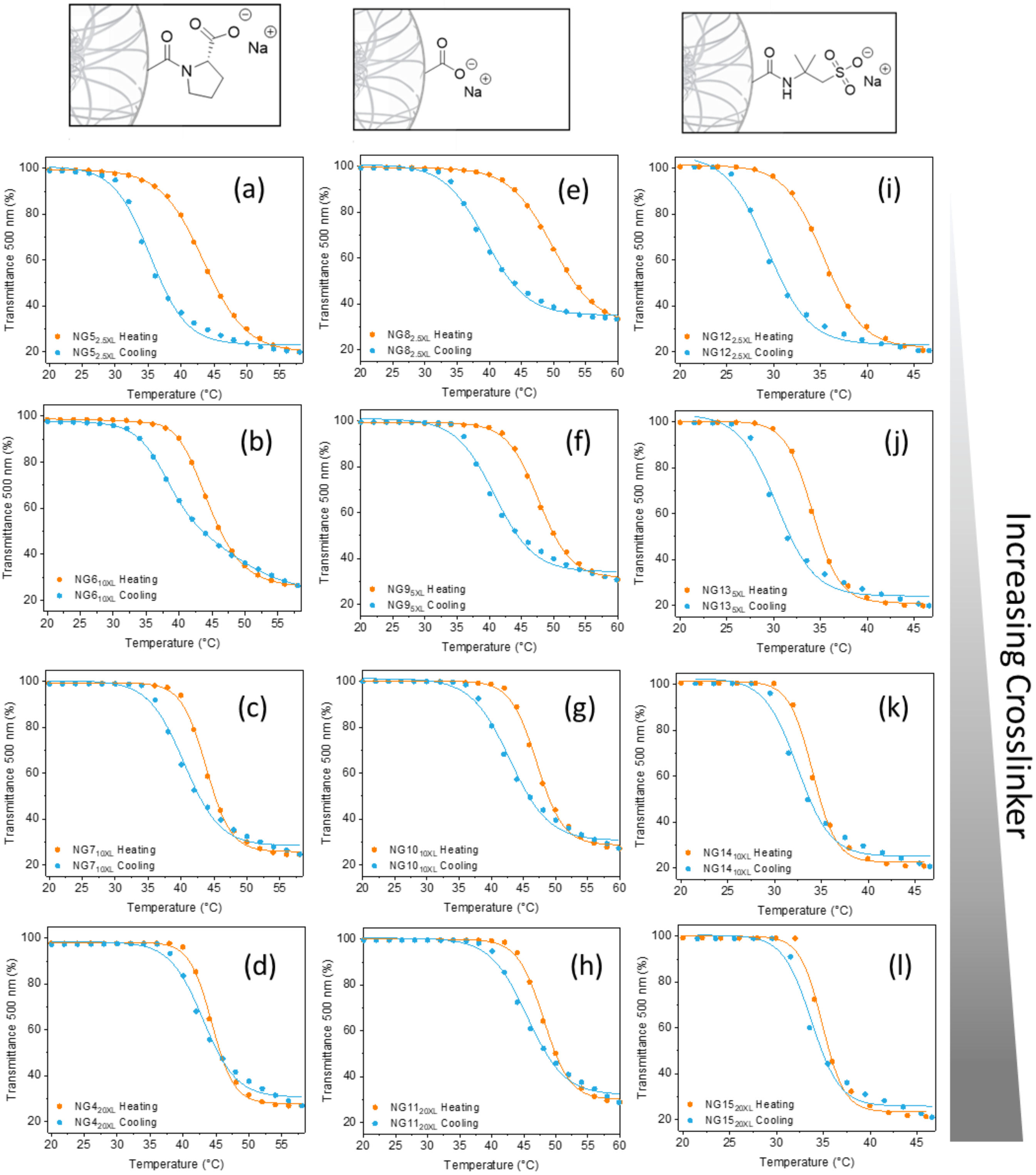

Table S2: Monomer conversion and total conversion data (^{1}H-NMR) of negatively charged nanogels.

NG	Monomer Conversion			
	NPAM	MBA	Negatively Charged Monomer	Total Monomer Conversion
	%			
	ProAM (2.5 molar%)			
$NG5_{2.5XL}$	92 ± 1	95 ± 8	>99	92 ± 1
$NG6_{5XL}$	88 ± 1	91 ± 1	>99	88 ± 1
$NG7_{10XL}$	91 ± 3	97 ± 1	>99	87 ± 6
$NG4_{20XL}$	86 ± 1	97 ± 1	>99	90 ± 1
	AA (2.5 molar%)			
$NG8_{2.5XL}$	91 ± 0	82 ± 1	>99	92 ± 2
$NG9_{5XL}$	91 ± 1	97 ± 5	>99	92 ± 1
$NG10_{10XL}$	92 ± 0	98 ± 2	>99	93 ± 0
$NG11_{20XL}$	92 ± 1	99 ± 1	>99	94 ± 1
	AMPS (2.5 molar%)			
$NG12_{2.5XL}$	90 ± 0	90 ± 3	>99	90 ± 0
$NG13_{5XL}$	83 ± 10	89 ± 1	>99	85 ± 9
$NG14_{10XL}$	88 ± 3	98 ± 1	>99	90 ± 1
$NG15_{20XL}$	83 ± 4	92 ± 5	>99	87 ± 2

Figure S5. Impact of nanogel-Lyso interactions on the VPTT of nanogels incorporating either ProAM (a), AA (b), or AMPS (c) as negatively chraged co-monomers, and containing either 2.5 molar% (left) or 20 molar% (right) XL. The raw data (dots) were fitted (line) using a sigmoidal curve fit (Boltzmann fit, OriginPro 2019) to obtain the inflection point of the curve (VPTT). First derivative graphs obtained from the raw transmittance plots are also displayed below them.

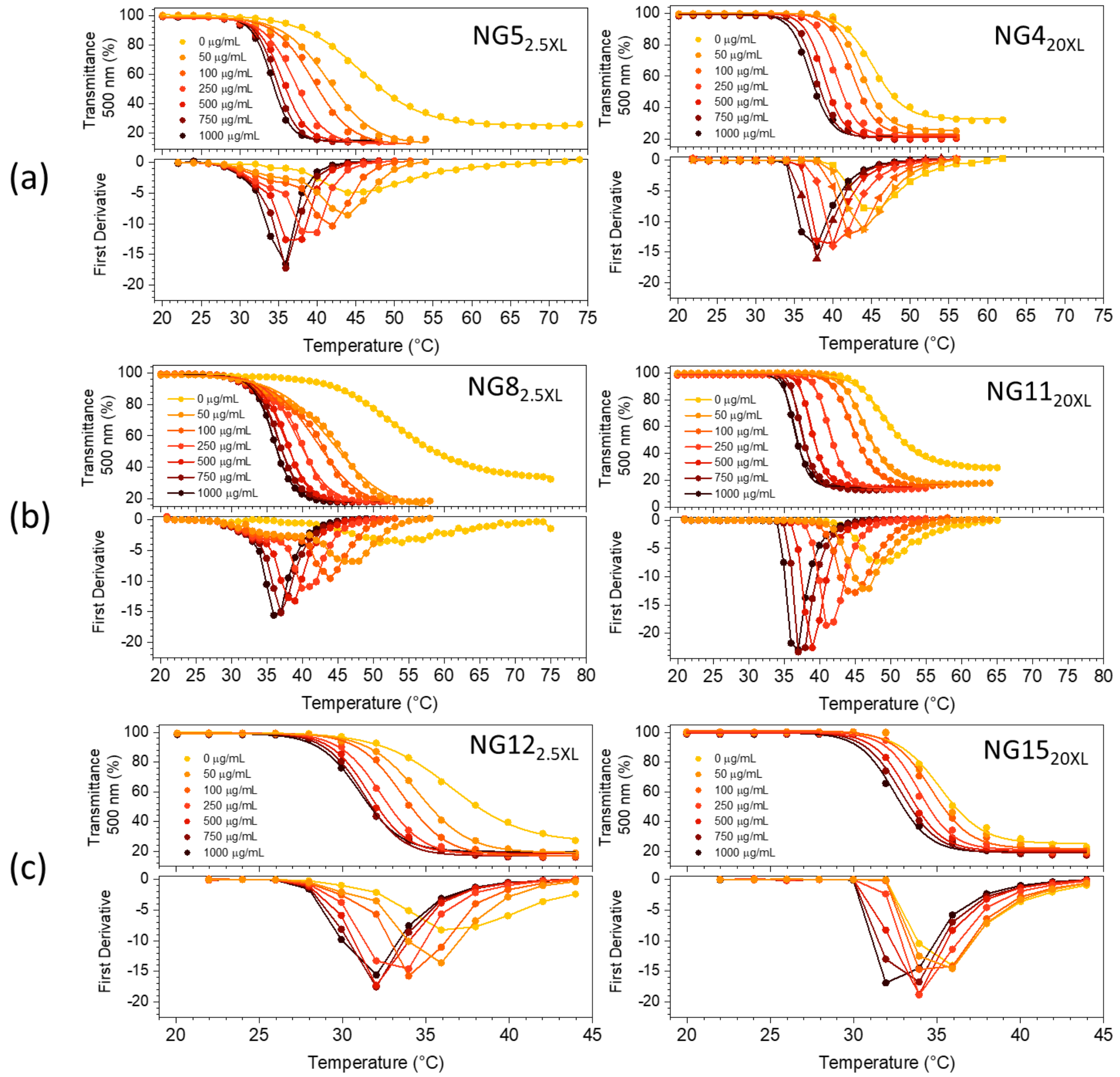

Table S3. Effect of Lyso addition (0 – 1000 µg/mL) on the VPTT (°C) values of NPAM-based nanogels incorporating either ProAM, AA, or AMPS as negatively charged monomers and with either 2.5 molar% or 20 molar% MBA.

NG	MBA (molar %)	Negatively Charged Monomer		Lyso concentration (µg/mL)						
				0	50	100	250	500	750	1000
NG5 2.5XL	2.5	ProAM	VPTT (°C)	44.2 ± 1.2	41.6 ± 0.3	39.9 ± 0.5	37.6 ± 0.7	35.7 ± 0.3	35.2 ± 0.4	34.6 ± 0.4
NG4 20XL	20.0			44.8 ± 0.4	43.5 ± 0.3	42.4 ± 0.2	40.2 ± 0.2	38.5 ± 0.3	37.6 ± 0.2	36.8 ± 0.3
NG8 2.5XL	2.5	AA		48.6 ± 1.6	43.3 ± 1.4	41.2 ± 1.1	38.5 ± 1.3	36.8 ± 0.8	35.9 ± 0.7	35.3 ± 0.7
NG11 20XL	20.0			49.1 ± 0.6	47.0 ± 0.6	45.2 ± 0.8	42.7 ± 1.2	40.3 ± 1.2	38.4 ± 1.0	37.7 ± 1.1
NG12 2.5XL	2.5	AMPS		35.9 ± 0.5	34.3 ± 0.3	33.3 ± 0.4	32.3 ± 0.4	31.4 ± 0.4	31.0 ± 0.3	30.7 ± 0.3
NG15 20XL	20.0			36.5 ± 1.5	36.7 ± 1.5	36.3 ± 1.4	35.5 ± 1.3	34.7 ± 1.3	34.4 ± 1.3	34.0 ± 1.3

Figure S6: Transmittance – temperature plots of negatively charged nanogels in the presence of increasing amounts of Lyso over a temperature range of 20 – 45 °C. Labels on the left side of the plots indicate the code of each nanogels as well as their monomer composition. Red-shaded plots on the left refer to data acquired while heating the sample, while blue-shaded plots on the right refer to data acquired while cooling the sample. Given that all samples present 100% transmittance at the beginning of the experiment, values of transmittance < 100% at the end of the cooling ramp are interpreted as sign of irreversible thermoresponsive transition.

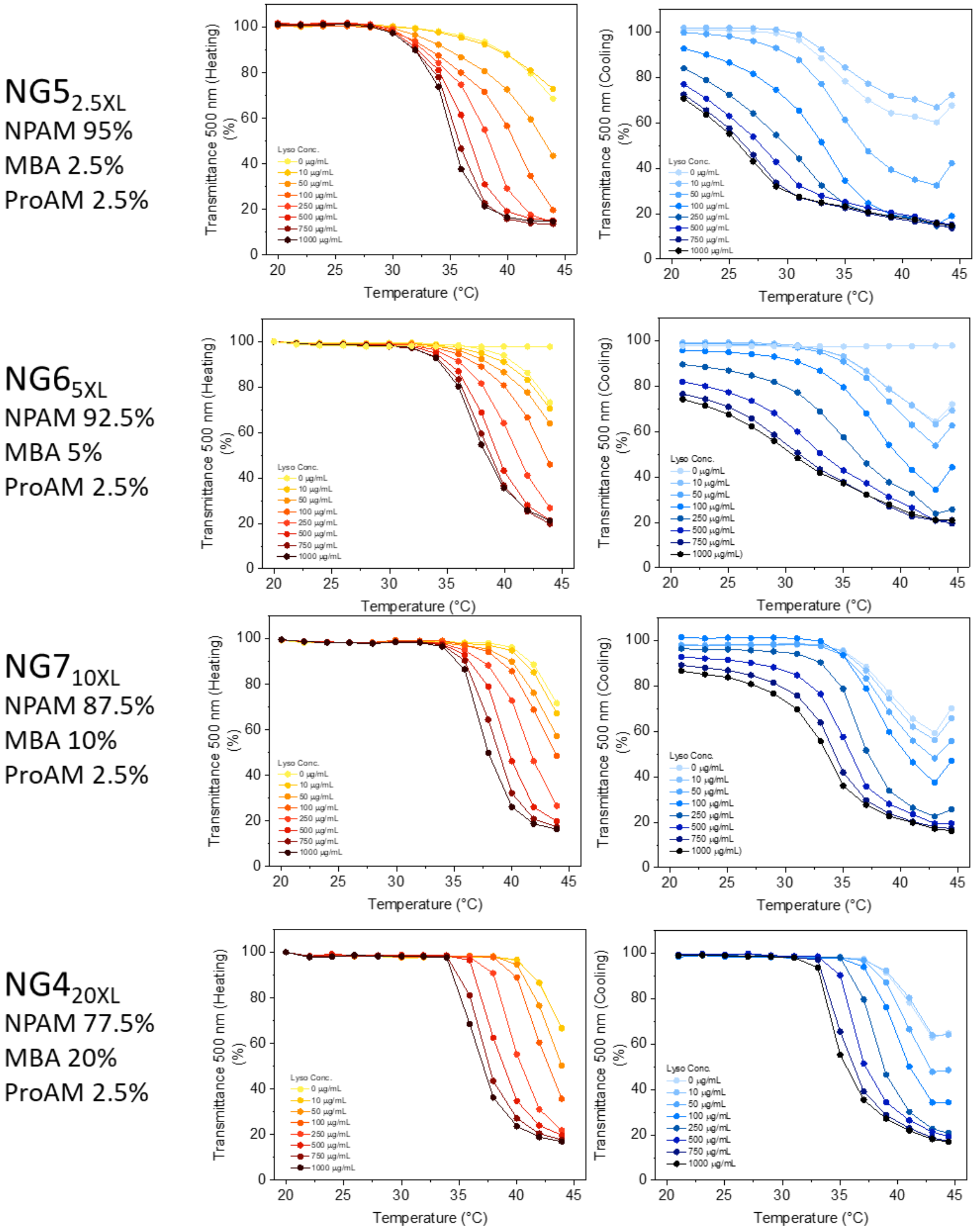

Figure continues in the following page.

NG8$_{2.5XL}$
NPAM 95%
MBA 2.5%
AA 2.5%

NG9$_{5XL}$
NPAM 92.5%
MBA 5%
AA 2.5%

NG10$_{10XL}$
NPAM 87.5%
MBA 10%
AA 2.5%

NG11$_{20XL}$
NPAM 77.5%
MBA 20%
AA 2.5%

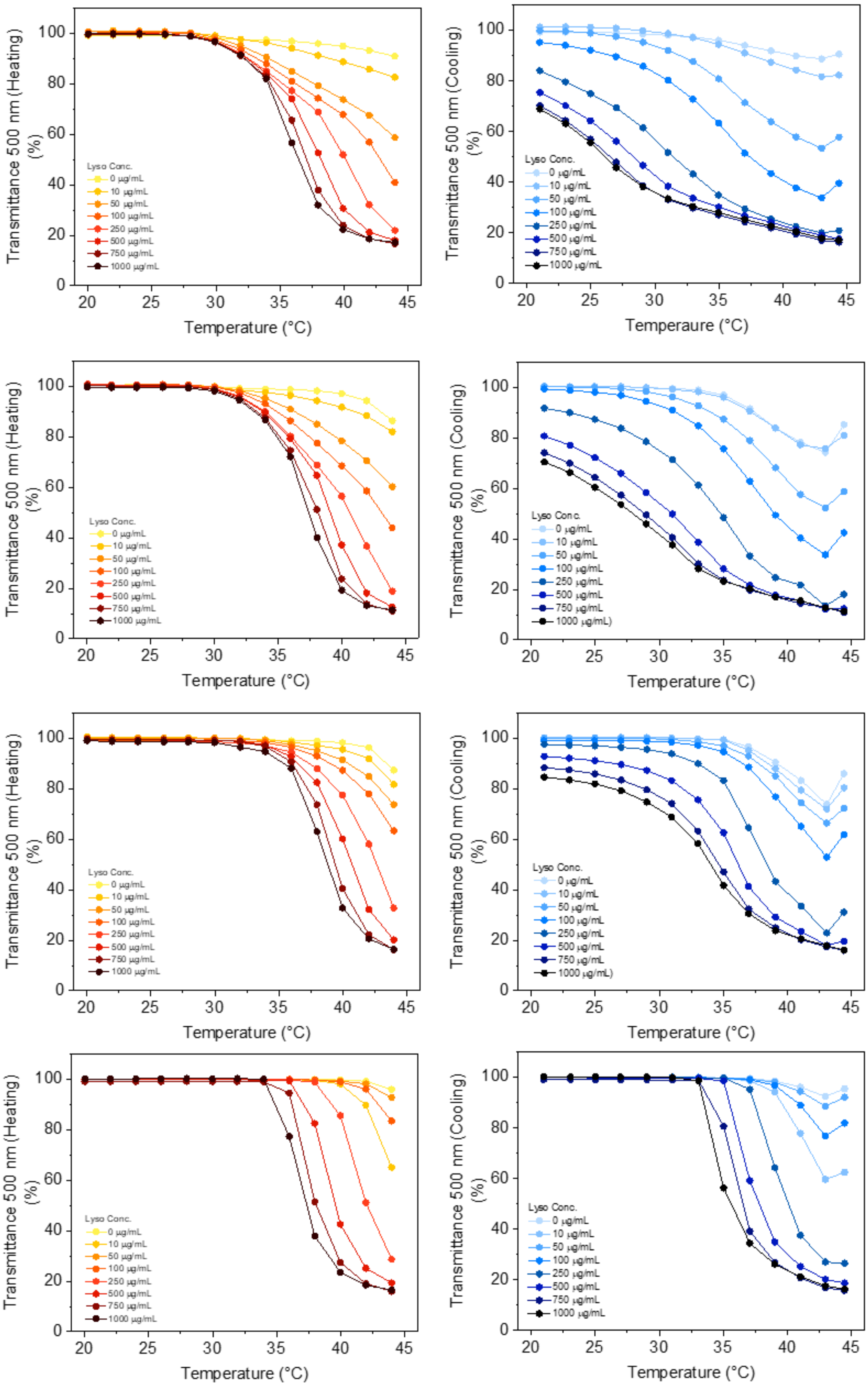

Figure continues in the following page.

NG12$_{2.5XL}$
NPAM 95%
MBA 2.5%
AMPS 2.5%

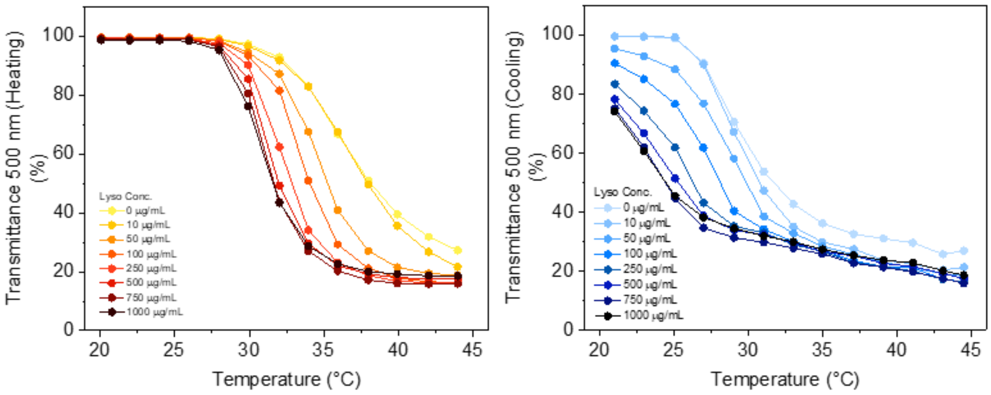

NG13$_{5XL}$
NPAM 92.5%
MBA 5%
AMPS 2.5%

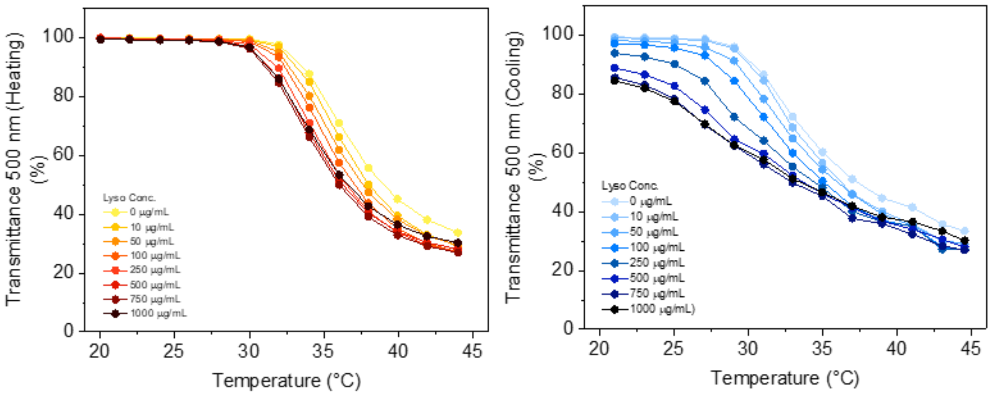

NG14$_{10XL}$
NPAM 87.5%
MBA 10%
AMPS 2.5%

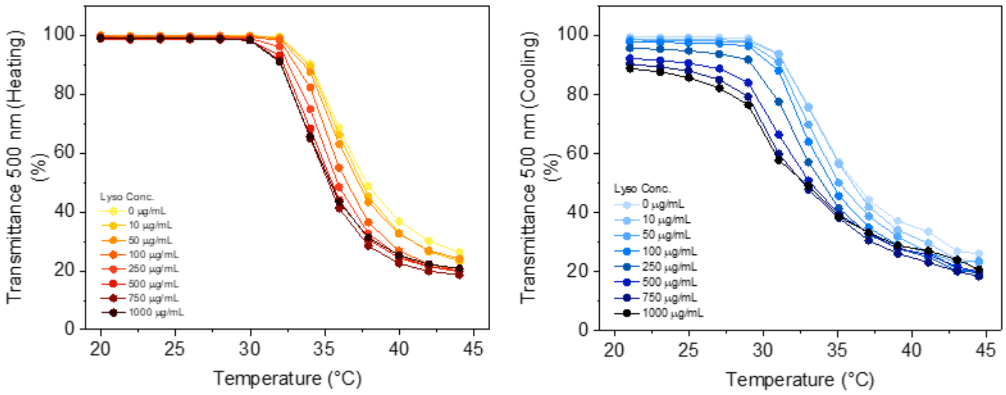

NG15$_{20XL}$
NPAM 77.5%
MBA 20%
AMPS 2.5%

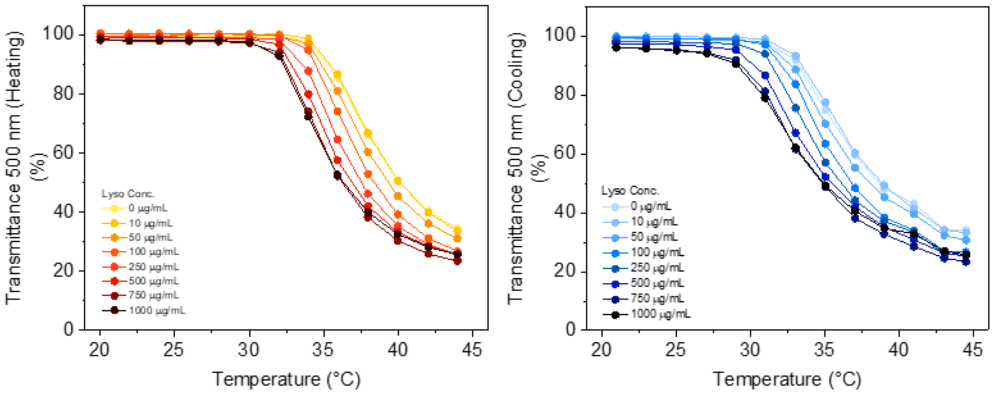

Figure S7. Dynamic light scattering measurements (intensity distribution) of $NG5_{2.5XL}$ and $NG4_{20XL}$ solutions in PBS (0.1 mg/mL) as a function of temperature. Samples were heated from 20 °C to 45 °C, and then back to 20 °C with a 2 °C step. Each measurement was taken after 10 minutes of equilibration time to allow the sample to reach a stable temperature.

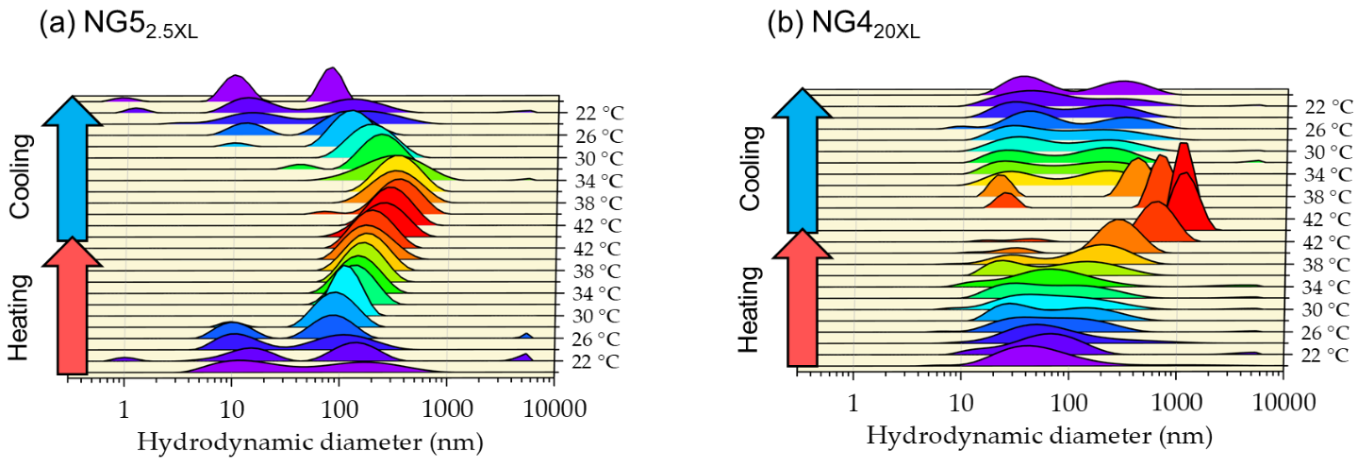

Figure S8: Circular dichroism of Lyso before and after a heating/cooling cycle (20 → 45 → 20 °C) and with and without negatively charged nanogels (ProAM).

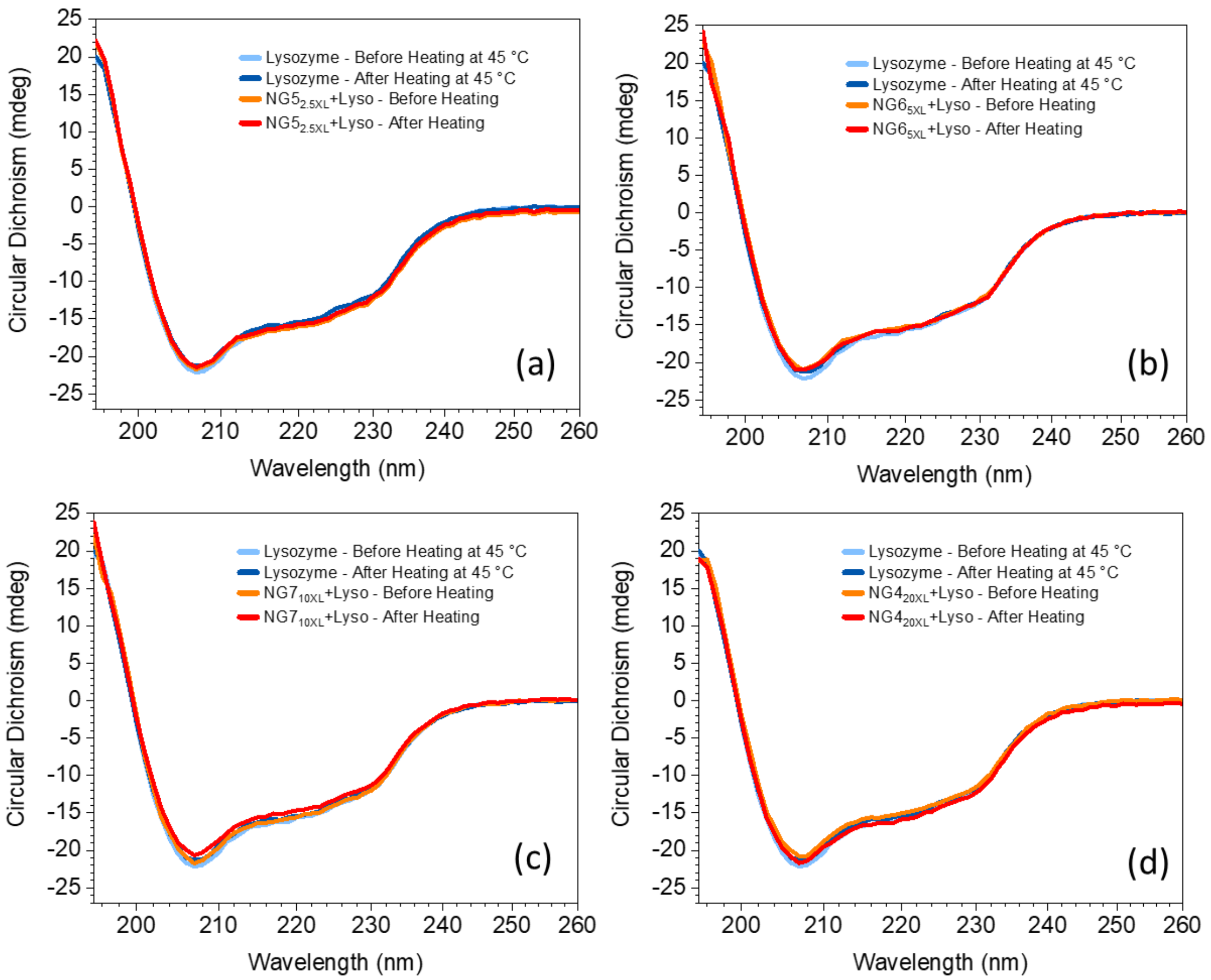

Table S4. Impact of Lyso (1 mg/mL) on the thermal hysteresis of 20 molar% crosslinked nanogels.

Nanogel	Hysteresis of nanogel alone (°C)	Hysteresis of nanogel + Lyso (°C)
$NG4_{20XL}$ (ProAM)	1.1 ± 0.4	1.1 ± 0.3
$NG11_{20XL}$ (AA)	2.7 ± 0.2	1.7 ± 0.4
$NG15_{20XL}$ (AMPS)	1.7 ± 0.4	1.6 ± 0.2
